# Detecting past and ongoing natural selection among ethnically Tibetan women at high altitude in Nepal

**DOI:** 10.1371/journal.pgen.1007650

**Published:** 2018-09-06

**Authors:** Choongwon Jeong, David B. Witonsky, Buddha Basnyat, Maniraj Neupane, Cynthia M. Beall, Geoff Childs, Sienna R. Craig, John Novembre, Anna Di Rienzo

**Affiliations:** 1 Department of Human Genetics, University of Chicago, Chicago, Illinois, United States of America; 2 Oxford University Clinical Research Unit, Patan Hospital, Kathmandu, Nepal; 3 Mountain Medicine Society of Nepal, Kathmandu, Nepal; 4 Department of Anthropology, Case Western Reserve University, Cleveland, Ohio, United States of America; 5 Department of Anthropology, Washington University in St. Louis, St. Louis, Missouri, United States of America; 6 Department of Anthropology, Dartmouth College, Hanover, New Hampshire, United States of America; University of Utah, UNITED STATES

## Abstract

Adaptive evolution in humans has rarely been characterized for its whole set of components, i.e. selective pressure, adaptive phenotype, beneficial alleles and realized fitness differential. We combined approaches for detecting polygenic adaptations and for mapping the genetic bases of physiological and fertility phenotypes in approximately 1000 indigenous ethnically Tibetan women from Nepal, adapted to high altitude. The results of genome-wide association analyses and tests for polygenic adaptations showed evidence of positive selection for alleles associated with more pregnancies and live births and evidence of negative selection for those associated with higher offspring mortality. Lower hemoglobin level did not show clear evidence for polygenic adaptation, despite its strong association with an *EPAS1* haplotype carrying selective sweep signals.

## Author summary

The adaptations to high altitude environments in Tibetan populations have long been highlighted as an important case study of adaptive evolution in our species. Recent genetic studies found two genes, *EGLN1* and *EPAS1*, the genetic variants in which were swept to high frequency in Tibetans due to strong positive natural selection. However, it still remains unclear if and how these and other genetic variants are connected to adaptive phenotypes and ultimately to fitness advantage. In this study, we collected genotype and phenotype information of 1,000 ethnically Tibetan women from the high Himalayan valleys in Nepal. Using both genome-wide association analysis and test for polygenic adaptations, we show that natural selection systematically altered frequency of alleles associated with reproductive outcomes to the direction of increasing fitness. That is, alleles associated with more pregnancies and live births, as well as those associated with lower offspring mortality, were under positive selection. Omitting the *EPAS1* haplotype under selective sweep, the other variants associated with lower hemoglobin did not collectively show a clear signal for polygenic adaptation. Our study highlights the polygenic nature of human adaptive traits.

## Introduction

Understanding the impact of natural selection on phenotypic variation has been a central focus of evolutionary biology since its beginning as a modern scientific discipline. Decades of research have accumulated evidence for widespread adaptive phenotypic evolution in nature, including correlations between phenotypes and environmental factors [1–3], and higher reproductive success of native individuals compared to visitors [4]. Beyond the phenotypic studies, much effort has been devoted, especially in humans, to identifying adaptive alleles through indirect statistical approaches that use genetic variation data and that can detect the impact of past selective pressures [5]. The most widely used family of approaches aims at detecting new beneficial mutations that were quickly driven to high frequency or fixation by natural selection, a model that is often referred to as selective sweep and that is likely to apply to adaptive alleles of large effect [5–9]. However, genome-wide association studies have revealed that most phenotypic variation in humans is highly polygenic; in other words, it is due to the combined effects of a large number of alleles with small effects [10–12]. Under this scenario, adaptations will tend to generate upward shifts in the frequency of adaptive alleles at many loci rather than a major shift at one or few loci, as is the case, for example, for lactase persistence. Methods for detecting polygenic adaptations combine two sources of information: genome-wide association studies (GWAS) provide alleles associated with a phenotype of interest as well as their effect size, and the population frequency of GWAS alleles enable inter-population comparison [13–15]. These indirect methods can provide evidence for past selective events, but each is sensitive to different selection models [16, 17], thus providing insights into a subset of adaptive alleles [18]. Moreover, these approaches cannot distinguish among selective effects on different fitness components, e.g. fertility vs. viability. A major advantage of indirect approaches is that they can detect selective sweep signals due to plausible, low selection coefficients (as long as *4N*_*e*_*s* > 1) with comparatively small sample sizes.

A complementary set of approaches aims at assessing directly the effects of genotype on reproductive fitness [19]. These direct approaches have many advantages, mainly the ability to detect selective events occurring in the present generation and the similar sensitivity to different selection models, e.g. balancing vs. directional selection [20]. However, they require large sample sizes to detect plausible selective coefficients. Large cohorts with genetic information are becoming increasingly available for humans, enabling approaches that were not feasible until recently [21, 22]. For example, a recent study analyzed two cohorts, for a total of more than 175,000 individuals, to assess genetic effects on viability by identifying alleles that consistently changed in frequency with age [23]. Another direct approach is to search for variants influencing fitness through GWAS of reproductive traits such as number of children ever born [24], twinning rate or mother’s age at first birth [25]. However, the genetic bases of reproductive traits remain markedly understudied, despite their great evolutionary and biomedical significance [26].

High altitude populations have emerged as an ideal system to study the genetic architecture of human adaptations. Populations of the high-altitude regions of Tibetan, Andean, and East African Plateaus have been exposed to the stress of hypobaric hypoxia for sufficient time [27] to have allowed the evolution of new adaptive traits [28–32]. Recent population genomic studies of Tibetans detected strong selective sweep signals in Tibetans at two loci, *EGLN1* (egl-9 family hypoxia inducible factor 1) and *EPAS1* (endothelial PAS domain containing protein 1) [33–35], each coding for a key component of the regulatory program responding to variation in oxygen supply [36]. Importantly, alleles in these genes that occur at high frequency in Tibetans but are rare elsewhere were also reported to be associated with lower hemoglobin concentration (Hb; g/dL) [33–35, 37] (but see [37–39]), consistent with many observations that unelevated Hb is characteristic of high-altitude Tibetans (reviewed in [32]).

Because the impact of hypobaric hypoxia on human physiology cannot be modified through behavioral or cultural practices, indigenous high altitude populations provide a rare opportunity to observe human evolution in action. Here, we took advantage of this property to design a study aimed at comprehesively dissecting adaptations to high altitude using both direct and indirect approaches. Our goal was to map, in the same sample, physiological as well as reproductive variables and to apply polygenic adaptation tests using information about the alleles associated with these traits. To this end, we collected genotype and phenotype data in a sample of ethnically Tibetan women in post-reproductive age (so that they had completed their family size). We found a single genome-wide significant association signal for oxygenated hemoglobin (“oxyHb”) at the *EPAS1* locus and several signals for reproductive traits. We tested for selective events that took place in the past, through indirect approaches that can detect polygenic adaptations, as well as for ongoing events, through the direct approach of mapping measures of reproductive success. We detected signatures of polygenic adaptation for reproductive traits such as numbers of livebirths and offspring mortality, consistent with selective processes that are still ongoing in contemporary populations. In contrast, we found little evidence for polygenic adaptations toward lower Hb.

## Results

### Genetic variation data of indigenous high-altitude individuals

In the context of studies of high-altitude adaptation, the term “Tibetan” refers to the modern descendants of the ancient indigenous population of the Tibetan plateau who share cultural and biological affinities and reside in several polities, including Nepal. To investigate the genetic bases of high-altitude adaptations in Tibetan populations, we collected physiological and reproductive phenotype data and saliva samples of 1,008 indigenous ethnically Tibetan women living at 3,000–4,000 m in the Mustang and Gorhka districts of Nepal (see Materials & Methods). All Tibetan participants were chosen to be 39 years of age or older so that their recorded reproductive history would have minimal confounding due to unrealized reproduction. We also obtained saliva samples for DNA extraction and analysis from 103 Sherpa participants (including ten parents and offspring trios) from the high-altitude regions in the Khumbu district in Nepal. The Sherpa data were included in the reference panel for genotype imputations and in the polygenic adaptation tests, but not in GWAS (see below).

**Fig 1:**
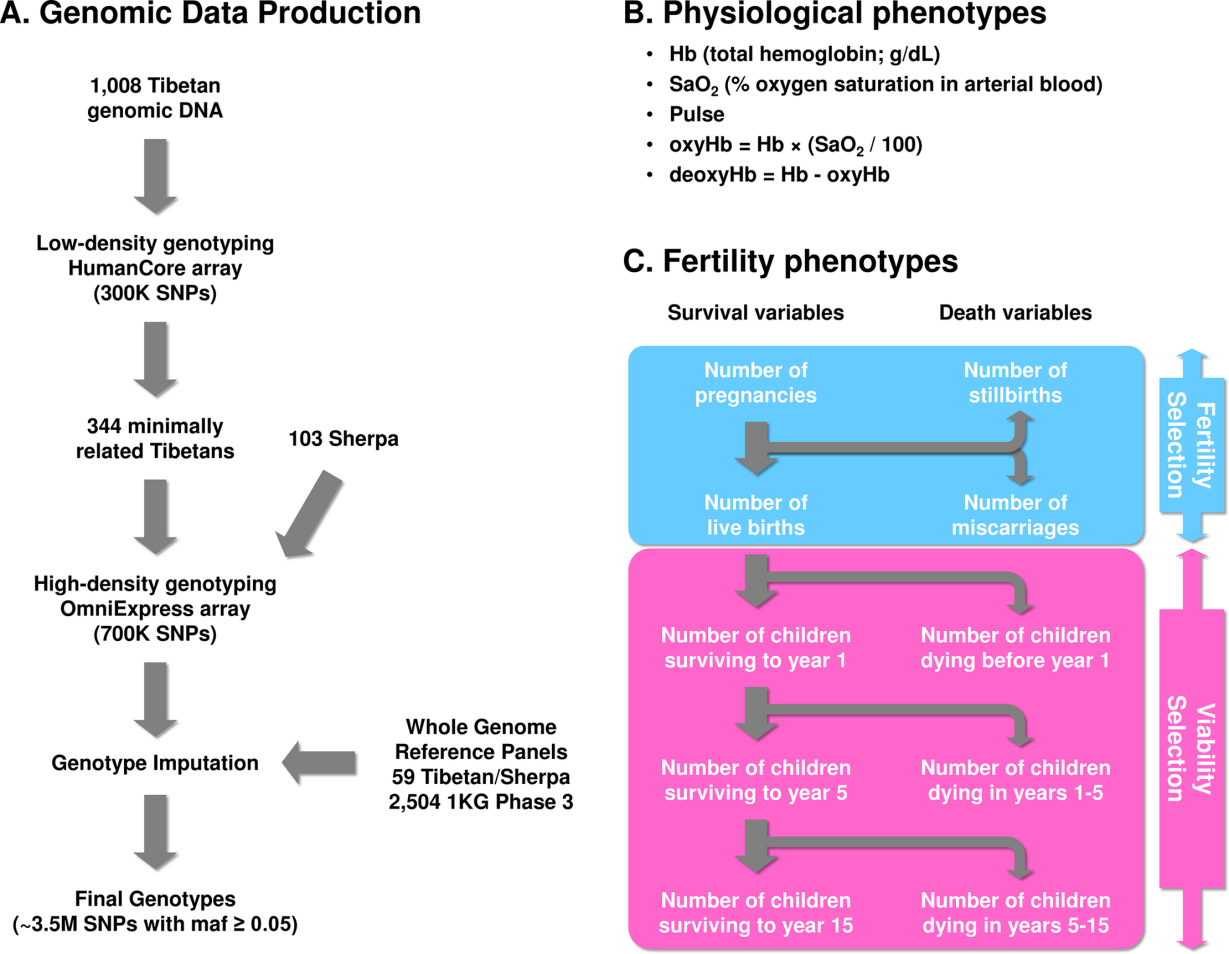
A schematic summary of the genotype and phenotype data of ethnic Tibetans in this study. (A) We array genotyped all individuals in several Illumina platforms and generated whole genome sequences for a representative subset without recent admixture. Then, all individuals went through genotype imputation using our high altitude sequence data (“high altitude panel”) and world-wide data (“1KG phase 3 panel”) as reference haplotype panels. (B) Three physiological phenotypes were directly measured in the field, and two additional ones (oxyHb and deoxyHb) were constructed from them. (C) Fertility phenotypes capture both fertility and viability selection components. For details, please see Materials & Methods.

Genetic variation data of our study participants were generated by a combination of experimental and computational tools (Fig 1; also see Materials & Methods). First, we generated novel genotype data for all participants using Illumina genotyping array platforms in multiple phases (S1 Table). Briefly, all Tibetans were first genotyped for about 300K markers on the HumanCore array with additional 2,553 custom markers to cover candidate regions, including the *EGLN1*, *EPAS1*, *HIF1A* (hypoxia inducible factor 1 alpha subunit) and *NOS2* (nitric oxide synthase 2) genes. Then, we genotyped a subset of 344 unrelated Tibetans (allowing up to first cousins) and all 103 Sherpa for over 700K markers on the OmniExpress array; the same individuals were separately genotyped for two nonsynonymous SNPs in the *EGLN1* gene, rs12097901 and rs186996510 [40]. Analyzing all Tibetan individuals together with published genome-wide genotype data of world-wide ancient and modern populations, we show that these Nepali Tibetans are genetically most closely related to other high altitude East Asians, such as Sherpas [41] and Tibetans from Lhasa [42], that they can be modeled as a mixture of Sherpas and South and Central Asians (e.g. Pathans), and that they derive on average only 3.4% of their ancestry from the latter gene pool (ranging 0.0–11.6%; S1 Text).

To augment publicly available reference panels for genotype imputation, we generated whole genome sequence data of 18 Sherpa and 35 Tibetans (S1 Table). Three Sherpa trios and four Tibetan mother-daughter duos were sequenced to high coverage (~ 20x), while the remaining 36 individuals were genetically unrelated and sequenced to low coverage (~ 5x, S1 Table). For sequencing, we chose Tibetan and Sherpa individuals with no signature of recent admixture (See Materials & Methods). Adding six previously published Tibetan and Sherpa genomes [41, 43], we obtained phased genotypes of 59 individuals including 9,742,498 variants, of which 1,364,150 were not found in the 1000 Genomes Project (1KGP) phase 3 data set [44]. Among the non-1KGP variants, 540,218 were included in dbSNP150 database, while 823,932 were not. Variant annotation using the ANNOVAR program [45] identified 8,679 nonsynonymous variants, 235 nonsense coding variants, and 126 splicing variants not present in the 1KGP (S2 Table). Among the non-1KGP variants, 29.46% and 24.14% occurred as singletons and doubletons, but 11.06% of them occurred at frequency 10% or higher (S2 Table). Using both the 1KGP phase 3 data and our high altitude sequence data as reference panels, we performed genotype imputation of all samples using the IMPUTE2 program [46] to generate the analysis-ready genotype data of 991 individuals covering about 3.5 million SNPs in Tibetans (see Materials & Methods).

### GWAS of fertility and physiological phenotypes in Tibetans

We performed GWAS of 23 phenotypes characterizing the reproductive history of our study participants using a linear mixed model-based approach as implemented in GEMMA [47]. Although they are partially correlated, these traits provide a comprehensive assessment of reproductive fitness and, importantly, allow evaluating the effects of selection on viability and fertility components, separately. We grouped our fertility phenotypes into two categories, “fertility counts” (e.g. number of live births) and “fertility proportions” (e.g. proportion of live births among pregnancies) (Table 1 and S3 Table describe the sample and summarizes the reproductive phenotypes). While the count phenotypes are more directly related to evolutionary fitness, they may be confounded by compensatory reproduction in case of a negative pregnancy outcome or by sociocultural factors that influence the count [48]. In contrast, the proportional variables are less affected by such factors and may provide information on the specific phase of the reproductive process affected by the associated genetic variation. Therefore, the counts and proportions may capture different aspects of the reproductive outcome. The GWAS was performed on the entire sample and on a subset referred to as continuously married (CM), that was composed of about 60% of participants who had been in a marital relationship throughout the ages of 25 to 40 (see Materials & Methods). This subset controls the variance in marital relationship status; on the other hand, the resulting smaller sample size reduces the power to detect significant associations.

**Table 1:** A summary for GWAS phenotypes and covariates for 991 Tibetan individuals included in our GWAS.

Phenotype	Mean	Median	SD^a^	Min	Max
**1. Physiological phenotypes**					
Hb	13.91	13.90	1.42	8.80	18.70
SaO_2_	87.36	88.00	4.45	72.00	96.00
Pulse	73.91	73.00	10.68	49.00	110.00
oxyHb	12.14	12.19	1.28	7.53	17.02
deoxyHb	1.77	1.66	0.69	0.38	4.70
**2. Fertility phenotypes**^b^					
# of pregnancies	5.67 (6.50)	6 (7)	2.87 (2.75)	0 (0)	15 (15)
# of live births	5.38 (6.24)	5 (6)	2.79 (2.64)	0 (0)	14 (13)
# of children born alive but died < 1 yr	0.83 (1.01)	0 (1)	1.15 (1.28)	0 (0)	8 (8)
# of children surviving at 1 yr but died < 5 yr	0.40 (0.47)	0 (0)	0.76 (0.85)	0 (0)	5 (5)
# of children surviving at 5 yr but died < 15 yr	0.26 (0.32)	0 (0)	0.59 (0.65)	0 (0)	3 (3)
# of children born alive but died < 5 yr	1.25 (1.51)	1 (1)	1.41 (1.53)	0 (0)	8 (8)
# of children born alive but died < 15 yr	1.57 (1.91)	1 (1)	1.63 (1.74)	0 (0)	8 (8)
# of children surviving at 1 yr	4.60 (5.27)	5 (5)	2.30 (2.21)	0 (0)	12 (12)
# of children surviving at 5 yr	4.14 (4.74)	4 (5)	2.11 (2.03)	0 (0)	12 (12)
# of children surviving at 15 yr	3.74 (4.23)	4 (4)	2.03 (2.03)	0 (0)	10 (10)
# of stillbirths	0.16 (0.15)	0 (0)	0.51 (0.47)	0 (0)	6 (5)
# of miscarriages	0.16 (0.16)	0 (0)	0.46 (0.46)	0 (0)	3 (3)
# of twin births	0.05 (0.05)	0 (0)	0.22 (0.22)	0 (0)	2 (1)
A woman’s age at her first childbirth	24.12 (22.39)	23 (22)	4.61 (3.14)	13 (13)	46 (43)
A woman’s age at her last pregnancy	36.99 (37.09)	38 (38)	6.00 (6.10)	19 (19)	58 (58)
**3. Covariates**					
Age	54.73	54.00	10.68	39.00	91.00
Alt^c^	3630	3632	244	2982	4052
Ft^d^	85.03	86.00	5.81	65.00	98.00
Subr^e^	Nubri (128); Tsum (131); Upper (597) and Lower Mustang (135)				
Ct^f^	Never used (670); Previously used (145); Currently in use (166)				
CM^g^	Continuously married (593); Not continuously married (398)				

^a^ Standard deviation

^b^ Numbers in parenthesis are summary statistics for the continuously married subset

^c^ Altitude of residence

^d^ fingertip temperature (°F)

^e^ sub-district label

^f^ use of contraception

^g^ continuously married

**Fig 2:**
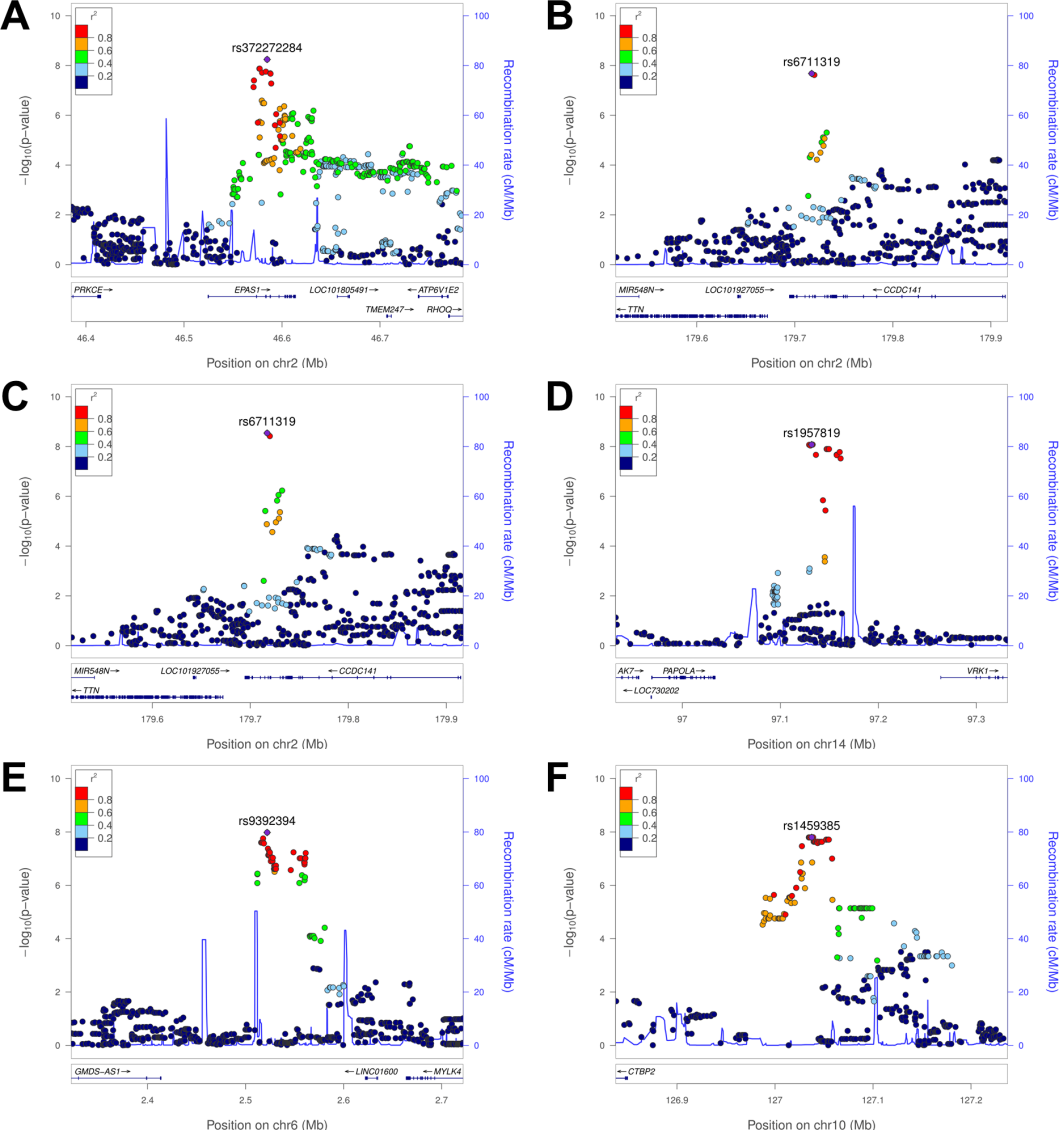
Locuszoom plots of the genome-wide significant associations found in Tibetans: (A) oxyHb and rs372272284 in the *EPAS1* gene, (B) the numbers of pregnancies or (C) live births and rs6711319 in the *CCDC141* gene, (D) the number of stillbirths and rs1957819 near the *PAPOLA* gene, (E, F) the proportion of offsprings died before age 15 years among the born alive and rs9392394/rs1459385. (A-C, E) are tests with all samples and (D, F) are those with the continuously married subset.

For the fertility count phenotypes, we found 55 SNPs with genome-wide significant genotype-phenotype associations, which can be reduced to six association peaks when linkage disequilibrium among associated SNP is taken into account (Table 2). These six peaks reflect five independent association signals, considering that the number of pregnancies and the number of live births are highly correlated. First, our analysis of three fertility count phenotypes yielded genome-wide significant association peaks. Two intronic SNPs in the *CCDC141* (coiled-coil domain containing 141) gene, with the top SNP rs6711319, were associated both with the number of pregnancies (*p* = 2.10×10^−8^) and with the number of live births (*p* = 2.89×10^−9^; Fig 2 and S1 Fig). Fourteen SNPs between the *PAPOLA* (poly(A) polymerase alpha) and the *VRK1* (vaccinia related kinase 1) genes were associated with the number of stillbirths in the continuously married subset (*p* ≥ 8.38×10^−9^; Fig 2 and S1 Fig). The same set of SNPs also showed a suggestive association in the complete sample set (“All”), including all individuals regardless of whether they were continuously married (*p* = 6.23×10^−5^ to 1.85×10^−4^). No expression quantitative trait loci (eQTLs) were detected in the GTEx Project data in this peak region [49], making it hard to connect the associated SNPs with a specific gene.

**Table 2:** Genome-wide significant association peaks among Tibetan women. “nSNPs” shows the number of genome-wide significant SNPs in each peak. “Top SNP” provides the rsID of the most significant SNP with effect / non-effect alleles. We chose the Tibetan minor allele as the effect allele. “Pos” is the genomic position of the top SNP in hg19 coordinates. Per allele effect size is provided in the *β* column. Continuously married (CM) subset phenotypes show the GWAS results restricted to about 60% of participants who stayed in marital relationship between age 25 and 40 (see Materials & methods).

Pheno	CHR	nSNPs	Top SNP	Pos	Minor allele frequency^a^	*β*^b^	*P*	PBS	Gene
oxyHb	2	8	rs372272284 (A/G)	46,584,859	0.248	0.386	5.71×10^−9^	1.05	*EPAS1* (genic)
# of pregnancies	2	2	rs6711319 (G/A)	179,717,217	0.248	-0.760	2.10×10^−8^	0.00	*CCDC141* (genic)
# of live births	2	2	rs6711319 (G/A)	179,717,217	0.248	-0.773	2.89×10^−9^	0.00	*CCDC141* (genic)
# of stillbirths (CM)	14	14	rs1957819 (T/C)	97,131,992	0.083	0.284	8.38×10^−9^	-0.01	*PAPOLA* (99 kb)
Proportion of children born alive who died < 15 yr	6	8	rs9392394 (G/A)	2,522,000	0.442	-0.318	1.05×10^−8^	0.00	*C6orf195* (101 kb)
Proportion of children born alive who died < 15 yr (CM)	10	29	rs1459385 (T/C)	127,037,417	0.089	0.704	1.59×10^−8^	0.02	*CTBP2* (188 kb)

^a^ Minor allele frequency in our Tibetan data set

^b^ Per minor allele effect size estimates are in the unit of g/dL (oxyHb), the number of children (the numbers of pregnancies, live births and stillbirths), or residuals in log-odds scale for fertility proportion phenotypes (proportion of children born alive who died < 15 yr)

For the fertility proportion phenotypes, two genome-wide significant association signals were detected (Table 2). Eight SNPs near *C6orf195*, with the top SNP rs9392394, were associated with the proportion of children who died before age 15 (*p* = 1.05×10^−8^); SNPs within 15 kb of this peak had been associated with heart, blood pressure and reticulocyte traits in GWAS [50–52]. Twenty-nine SNPs near *CTBP2*, with the top SNP rs1459385, were associated with the same phenotype in the continuously married subset (*p* = 1.59×10^−8^; Fig 2 and S1 Fig); other genes in this region include *TEX36* (testis expressed 36) and *EDRF1* (erythroid differentiation regulatory factor 1, which regualtes the expression of globin genes). No eQTLs were detected in the GTEx Project data in these association regions.

The genetic bases of Hb, percent of oxygen saturation of hemoglobin (SaO_2_), and pulse have been previously studied in outbred populations mainly of European ancestry [52–58]. Here, we performed GWAS of these key physiological phenotypes in Tibetans, measured by the same non-invasive device, to potentially uncover population-specific genetic determinants. We derived two additional composite phenotypes: oxygenated hemoglobin concentration (“oxyHb”, defined as the product of Hb and SaO_2_ divided by 100), and deoxygenated hemoglobin concentration (“deoxyHb”, defined as the difference between Hb and oxyHb). Consistent with findings from other studies, these women had an average hemoglobin concentration of 13.8 g/dL ± 1.3 g/dL (mean ± 1 standard deviation). Table 1 and S3 Table describe the sample and summarize the phenotypic data. Each GWAS included about 3.5 million SNPs with minor allele frequency (maf) ≥ 0.05.

Eight SNPs within a 17 kb intronic region of the *EPAS1* gene were significantly associated with oxyHb (*p* ≤ 5 × 10^−8^ for all eight SNPs, with the top signal at rs372272284; Table 2, Fig 2 and S2 Fig). Hb and oxyHb were strongly correlated (Pearson *r* = 0.874), and all eight SNPs were also strongly associated with Hb (*p* ≤ 4.10×10^−7^; S4 Table). This is the first report of an association of the derived Tibetan *EPAS1* alleles with a hemoglobin trait that reaches genome-wide significance levels. The results, including the estimated effect size of 0.332 g/dL per allele, support previous candidate gene studies for Hb [33, 34]. Due to strong linkage disequilibrium (LD), the signature of a selective sweep around the *EPAS1* gene in Tibetans extends farther than 100 kb; however, our large sample size and dense genetic variation data allowed us to narrow down the association signal to a 17 kb region.

Conditioning on the genotype of rs372272284, no residual association with either Hb or oxyHb was observed in the *EPAS1* locus (*p* ≥ 0.770; S3 Fig). This includes a previously identified “Tibetan-enriched” deletion (“TED”), 81kb downstream of the *EPAS1* gene, present in Tibetans but not in the introgressed Denisovan haplotype [59]. TED is in LD with the eight significant SNPs in our data set (Pearson *r* = 0.771–0.783), but its association with Hb and oxyHb was much weaker than our top SNPs (*p* = 1.64×10^−3^ and 3.09×10^−4^, respectively).

In contrast, we did not detect significant associations in the *EGLN1* gene, even when we added menopause status, a female-specific covariate of Hb, as an additional covariate or confined our analysis to post-menopausal women (*p* ≥ 0.641). Moreover, our data showed no significant interaction between the *EPAS1* and *EGLN1* SNPs (rs372272284 and rs186996510, respectively) in the association with Hb (marginal effect for the *EGLN1* SNP rs186996510, *p* = 0.196, effect size *β* = 0.095 ± 0.074 g/dL; interaction effect, *p* = 0.613; *β* = 0.057 ± 0.114) [60]. Our results in post-reproductive females are consistent with and reinforce recent evidence suggesting that if *EGLN1* SNPs affect Hb, they do so only in males (S2 Text) [37–39].

To see if the phenotype-associated markers are under strong positive selection, we used the population branch statistic (PBS) [34] with 1KGP phase 3 CHB (Han Chinese in Beijing, China) as a comparison group and 1KGP phase CEU (CEPH Utah residents with Northern and Western European ancestry) as an outgroup. Based on the allele frequency differentiation, PBS measures the level of allele frequency change specific to the target population (i.e. Tibetan) that is not shared by its comparison group. We find no selective sweep signal over the genome-wide significant association peaks except for *EPAS1*, and find no loci with strong signatures of selective sweep beyond *EPAS1* (PBS = 1.073, rs73926264) and *EGLN1* (PBS = 0.797, rs186996510; S3 Text and S5 Table).

### Hb and pulse are correlated with aspects of reproductive success in Tibetan

A previous analysis of this sample of Tibetan women found strong relationships between physiological traits and reproductive success in this Tibetan sample by using a large set of covariates, including physiological, sociocultural, and socioeconomic variables (e.g. relative wealth rank, type of marriage, and marital status) [48]. Because the genetic analyses performed here used only a subset of those covariates, we tested for association of physiological traits and reproductive success by correcting for the specific set of covariates used in our GWAS. Consistent with the previous analysis, we found that lower Hb (in females in post-reproductive years) correlated with a higher proportion of live births among pregnancies (*p* = 0.002). We also found that Hb correlated positively with the numbers of stillbirths or miscarriages (*p* = 0.040 and 0.057, respectively), as well as their proportions among pregnancies (*p* = 0.023 and 0.033 for stillbirths and miscarriages, respectively).

Another interesting finding was the negative correlations between pulse and most of the fertility phenotypes, with the strongest correlations found with the numbers of pregnancies and livebirths (*p* = 2.02×10^−5^ and 2.76×10^−5^, respectively; S6 Table). Pulse’s negative association with a woman’s age at her last pregnancy partially accounts for this strong correlation; however, the association between pulse and the number of pregnancies remained significant (*p* = 0.005) after correcting for a woman’s age at her last pregnancy, even if weaker. The pulse and fertility traits were previously shown to be marginally correlated if a larger set of physiological and sociocultural covariates was included in the model (*p* = 0.130 and 0.069 for the numbers of pregnancies and live births, respectively) [48].

These results suggest that both low Hb and low pulse are associated with better reproductive outcomes at high altitude and raise the possibility that genetic variants decreasing these traits were selected for in Tibetans.

### Polygenic adaptations for physiological and reproductive traits

The women’s reproductive history data offer a unique opportunity to ask if selective sweep signals are associated with ongoing selection in contemporary Tibetans due to maternal factors. Consistent with previous studies, the *EPAS1* and *EGLN1* loci harbored the highest PBS values (S5 Table). However, we did not detect an association between *EGLN1* and *EPAS1* SNPs and any of the direct measures of fitness; nominal levels of significance were observed in some cases, but no test reached significance after multiple test correction. Power calculations for the fertility count phenotypes suggest that we can detect such an association only if the associated selection coefficient is extremely high (≥ 6.6% per allele for 80% power given a single test; S7 Table) and well above previous estimates for both *EPAS1* and *EGLN1*, 1.5% and 2.9%, respectively [37, 61]. Therefore, these results do not rule out that these variants are advantageous. Other SNPs with high PBS values also did not show a significant association with fertility variables.

To test for polygenic adaptations for low Hb and low pulse, we used two methods specifically designed to detect consistent changes in the frequency of alleles at many independent GWAS SNPs for a trait of interest. The first approach considers the frequency difference of the trait-increasing alleles between pairs of populations, specifically Tibetans or Sherpa and 1KGP CHB; the results are compared to 10,000 sets of control SNPs [13]. The second, more recent approach [14] calculates a genetic value for a trait of interest in each population by summing up the product of the frequency at each GWAS SNP and the effect size of that SNP and it compares GWAS-ascertained SNPs with a large number of control SNPs. Specifically, we focused on two tests. The “overdispersion” test asks if allele frequencies of the GWAS SNPs as a group show either unusually big differentiation across populations or unexpectedly strong correlation in the direction of change. The “outlier” test asks if the genetic value of a trait in a population or a group of populations is significantly different from that of the other populations.

**Fig 3:**
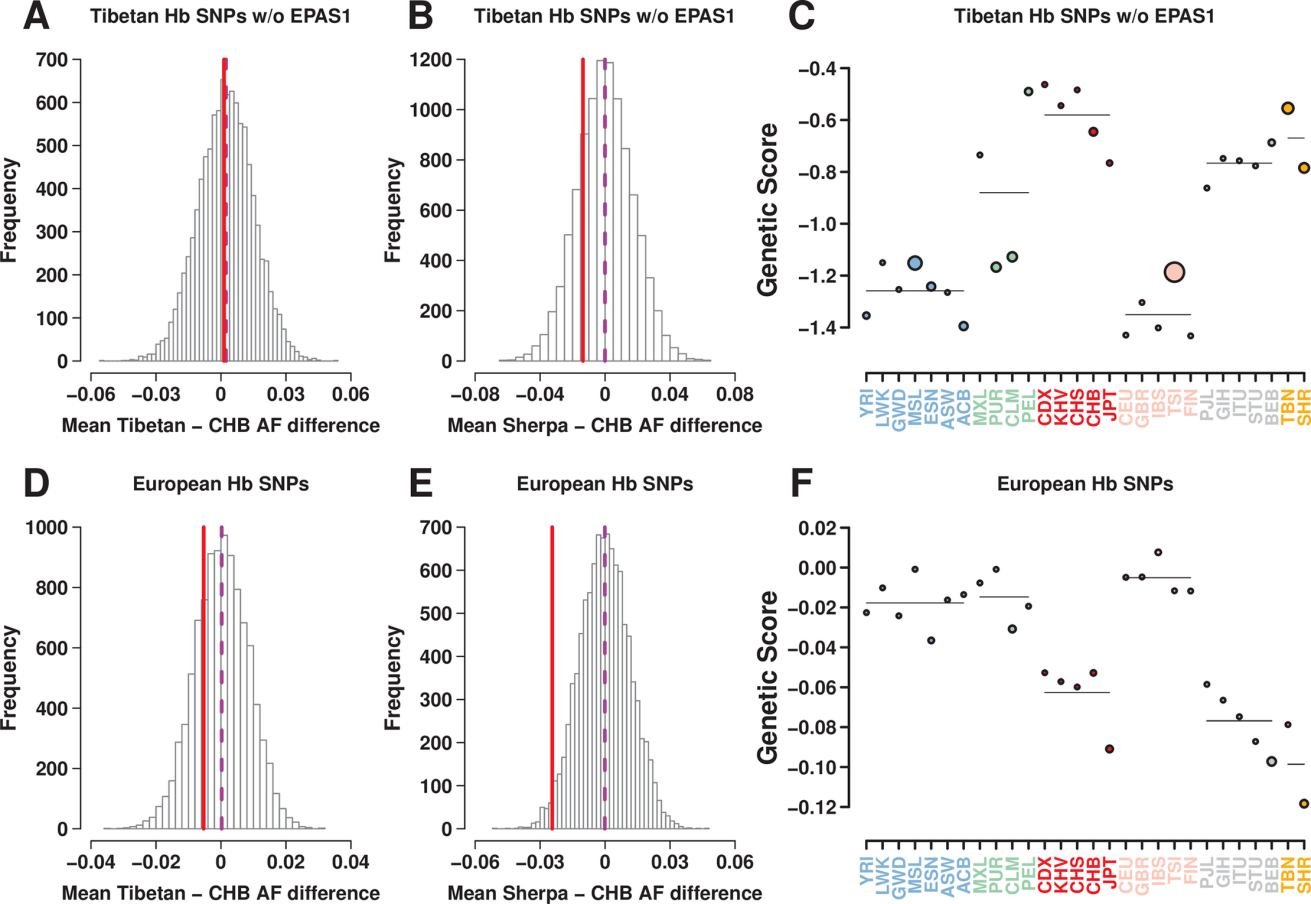
Tests of polygenic adaptation of Hb-associated SNPs: (A-C) 35 SNPs from our Tibetan GWAS (*p* ≤ 10^−4^) after excluding the *EPAS1* SNP rs372272284, and (D-F) 91 genome-wide significant SNPs from a large GWAS of mostly European cohorts. The mean frequency differences of trait-increasing alleles between Tibetans and CHB (A, D) and between Sherpa and CHB (B, E) were presented (solid red line) together with the empirical null distribution of 10,000 sets of matched random SNPs. (C, F) The genetic values of populations (filled dots) and of regions (horizontal lines) were plotted. The size of dots and the width of lines are proportional to the significance of the corresponding outlier test.

To test for positive selection for lower Hb, we used the 36 and 43 independent SNPs (*p* ≤ 10^−4^) ascertained from our Tibetan Hb and oxyHb GWAS, respectively. Compared to control SNPs [13], the Hb SNPs identified in Tibetans showed on average a lower frequency of Hb-increasing alleles in both Tibetans and Sherpa, suggesting selection favoring lower Hb levels (one-sided empirical-*p* = 0.047 and 0.018, respectively; Fig 3). The Tibetan oxyHb SNP set also showed a similar pattern (*p* = 0.102 and 0.046 for Tibetans and Sherpa, respectively; S4 Fig). However, when the *EPAS1* SNP rs372272284 was excluded, no difference between the Hb- or oxyHb-associated SNPs and control SNPs was observed (*p* ≥ 0.211; Fig 3 and S4 Fig). Thus, the overall frequency difference seemed entirely due to the large frequency differentiation of the *EPAS1* SNP: the Hb-increasing allele frequency was 0.253 and 0.990 for Tibetans and CHB, respectively.

GWAS SNP effect sizes have been shown to be correlated between European and East Asian populations [62], implying that at least part of the SNPs identified in the large Hb GWAS in Europeans are also likely to be associated with Hb in our Tibetan sample. Therefore, we also tested for polygenic adaptation using SNPs identified in a large European GWAS [52]; of the 140 European GWAS SNPs, we used the 91 SNPs that were called in our data set. This set of SNPs did not include any *EPAS1* SNPs, because the Tibetan *EPAS1* haplotype is virtually absent outside Tibetan populations [63]. We found a trend towards lower frequencies of Hb-increasing alleles in both Tibetan and Sherpa, but this trend reached nominal levels of significance only in Sherpa (*p* = 0.249 and 0.019 for Tibetans and Sherpa, respectively; Fig 3). Consistent with the results of the pairwise test, neither the overdispersion test nor the outlier test for the high-altitude populations yielded results reaching nominal levels of significance (*p*_overdispersion_ = 0.695 and *p*_outlier_ = 0.066 with no multiple test correction) for oxyHb, or Hb (*p*_overdispersion_ = 0.201 and *p*_outlier_ = 0.846 with no multiple test correction) when the *EPAS1* SNP was excluded (Fig 3, S4 Fig and S8 Table). Similar to the pairwise population test results, the outlier test was highly significant when the *EPAS1* SNP was included (*p* ≤ 0.0008; Fig 3, S4 Fig and S8 Table). Using the Hb associated SNPs from the European GWAS, we again observed a trend toward lower genetic values in the high altitude populations, but it did not reach levels of statistical significance (*p*_overdispersion_ = 0.433 and *p*_outlier_ = 0.110 with no multiple test correction). Therefore, these analyses do not provide evidence that alleles associated with lower Hb levels were selected for, except for the *EPAS1* locus. Given that the *EPAS1* SNPs explain a small fraction of the total variation in Hb levels (2.7% in our cohort), these results raise the question of whether unelevated Hb *per se* was the adaptive trait in Tibetans.

**Fig 4:**
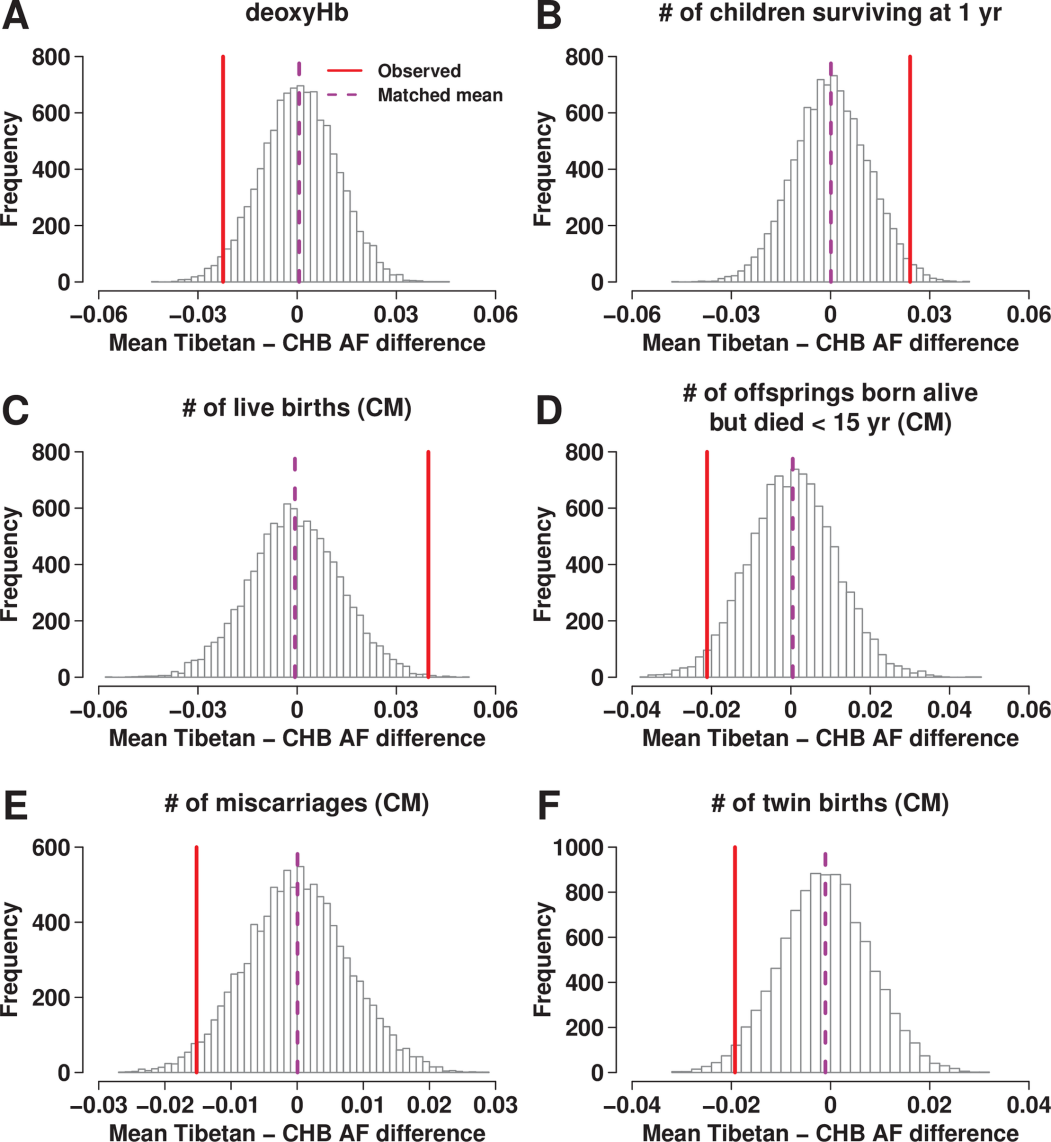
Signatures of polygenic adaptations in Tibetans shown for (A) deoxyHb, (B) the number of children surviving at 1 year, (C) the number of live births, (D) the number of children born alive but died before 15 years, (E) the number of miscarriages and (F) the number of twin births. The mean frequency difference of trait-increasing alleles was presented (solid red line) together with the empirical null distribution of 10,000 sets of matched random SNPs. (C-F) uses GWAS SNPs from the “CM” subset.

In contrast to Hb and oxyHb, deoxyHb showed significant polygenic adaptation signals, with both the outlier and the pairwise difference tests (*p*_outlier_ = 0.023 and *p*_pairwise_ = 0.002; Fig 4, S4 Fig and S8 Table). This result is compatible with the lack of evidence for polygenic adaptation toward lower Hb in post-reproductive females because deoxyHb is not strongly correlated with deoxyHb (*r* = 0.441 compared to that with oxyHb *r* = 0.874). The alleles associated with higher deoxyHb in Tibetans were on average less common in Tibetans than in 1KGP CHB and the genetic values of deoxyHb in Tibetans and the Sherpa tended to be lower than those in 1KGP East Asians. Maximizing oxygen delivery while minimizing blood viscosity is likely to be beneficial in high-altitude environments; therefore, this advantage may underlie our signal of polygenic adaptation for lower deoxyHb.

Based on 123 overlapping SNPs ascertained from a recently released large GWAS using the UK Biobank data [64], we find a very strong signal for low pulse in the pairwise difference test (*p*_*pairwise*_ = 0.0001; S5 Fig and S8 Table). Results using 52 SNPs ascertained from our Tibetan GWAS also show a marginally significant deviation in the same direction (*p*_*pairwise*_ = 0.0620). Interestingly, we do not see significant signals in either overdispersion or outlier test for Tibetans or Sherpa (*p* > 0.05); instead, we see a strong signal in the outlier test for lowland East Asians toward high pulse (*p*_*outlier*_ = 0.001; S5 Fig). The most parsimonious interpretation of these findings is that selection favored higher pulse only in low altitude East Asians, although other explanations are also possible. We did not find a significant result for any polygenic adaptation test for SaO_2_ (S8 Table).

The GWAS of reproductive traits allowed us to identify candidate variants that are currently being selected for in the sampled Tibetan population. In our results, none of the most strongly associated variants with reproductive outcomes showed strong signals of selective sweeps. However, if these variants affected reproductive fitness in the past in addition to the current generation, we might expect signals of polygenic adaptation. Indeed, a number of reproductive traits showed strong signatures of polygenic adaptations based on the outlier test; the pairwise population difference test, which uses less information and hence is likely to be less powerful, gives broadly consistent results, although at lower levels of significance (Fig 4, Table 3, S6 Fig and S8 Table). We see significant polygenic adaptation signals in several measures directly related to reproductive fitness. Interestingly, the significant signals are observed for both the viability (e.g. the number of children born alive but died before 15 years; *p*_outlier_ = 0.000, *p*_pairwise_ = 0.024) and the fertility fitness component (e.g. the number of live births; *p*_outlier_ = 0.002, *p*_pairwise_ = 0.002). Furthermore, consistent with expectations, alleles increasing offspring mortality were selected against whereas those increasing offspring survival were positively selected for. A variable known to be directly linked to reproductive fitness, i.e. a woman’s age at her first childbirth, is also under selection, with earlier ages being advantageous, as expected. Twinning appears to have been selected against in Tibetan women. Although twinning may increase fitness, it is also associated with increased risks to mother and offspring due to limits on women’s ability to support adequate weight gain for two babies during the third trimester and to the lower birth weight of twins relative to singletons [65], which in turn is associated with higher neonatal and infant mortality.

**Table 3:** Results of polygenic adaptation tests for a chosen set of physiology and fertility phenotypes. Results for all GWAS phenotypes are presented in S8 Table.

Phenotype	Direction^a^	GWAS sample^b^	Outlier test		*P*_frq.diff_^c^
			Statistic	*P*-value	
**1. Fertility phenotypes**					
A woman’s age at her first childbirth	-	CM	-1.9286	0.0000	0.576
# of live births	+	CM	2.5062	0.0020	0.002
# of miscarriages	-	CM	-3.0936	0.0000	0.024
# of children born alive but died < 1 yr	-	CM	-2.4325	0.0000	0.012
# of children born alive but died < 15 yr	-	CM	-2.0293	0.0000	0.024
# of children born alive but died < 5 yr	-	All	-1.5043	0.0000	0.134
# of children died ≥ 5 yr and < 15 yrs	-	CM	-1.1255	0.0256	0.304
# of children surviving at 1 yr	+	All	1.7603	0.0100	0.017
Proportion of children born alive but died < 15 yr	-	CM	-1.2639	0.0224	0.101
Proportion of stillbirths among pregnancies	-	CM	-2.1763	0.0008	0.230
Proportion of stillbirths among pregnancies	-	All	-1.6320	0.0004	0.285
# of twin births	-	CM	-2.4344	0.0000	0.021
# of twin births	-	All	-1.1421	0.0024	0.139
**2. Physiological phenotypes**					
Hb	-		-1.0490	0.0000	0.047
Hb without *EPAS1*	-		-0.5003	0.8460	0.467
oxyHb	-		-0.5657	0.0008	0.102
oxyHb without *EPAS1*	-		0.8596	0.0656	0.554
deoxyHb	-		-1.4628	0.0024	0.023
Pulse	-		-0.4412	0.4048	0.062

^a^ Assumed direction of positive selection for each phenotype

^b^ All GWAS data used in this table are from our Tibetan sample. Two sample sets for fertility phenotypes were used: all samples (All) and continuously married subset (CM)

^c^ One-sided empirical *p*-value for the mean frequency difference test

## Discussion

We identified several genome-wide significant associations with key physiological and fertility phenotypes in Tibetans (Fig 2 and Table 2), by analyzing new dense genome-wide variation data of over 1,000 indigenous inhabitants above 3,000 m in Nepal (2,982–4,052 m with mean of 3,630 m; S1 Table). Using genetic variants identified in our Tibetan GWAS, we found that several phenotypes showed signatures of polygenic adaptation towards better reproductive outcomes (e.g. the number of livebirths) (Table 3, S6 Fig and S8 Table). We also found evidence for polygenic adaptations for changes in pulse, possibly due to selection for higher pulse in low altitude East Asians that did not act on Tibetans and Sherpa. Surprisingly, we did not find clear evidence for polygenic adaptation towards low Hb in Tibetans beyond a link through the *EPAS1* gene, even though we confirmed a correlation between low Hb and better reproductive outcomes. Because Hb concentration is a polygenic trait, these results raise the question of whether lower hemoglobin is causally related to higher reproductive fitness.

The availability of reproductive history data in a population with little or no birth control offers unique opportunities for elucidating the adaptation process. Indeed, the ethnic Tibetan women sampled in this study have high birth rates (the number of livebirths = 5.38 ± 2.79; mean ± 1 standard deviation) and live in a mostly traditional society, where modern medical care, including in some regions contraception, has been introduced only very recently [48]. The reproductive data, collected in women who had largely completed their family size, allowed testing for a relationship between genetic or phenotypic variation and fitness differential. Genetic variation carrying well-established signals of selective sweeps, i.e. *EGLN1* and *EPAS1* SNPs, was not significantly associated with reproductive success probably due to low power: we estimated that the lowest selection coefficients that we had 80% power to detect were 6.6% and 7.4% at the *EGLN1* and *EPAS1* SNPs, respectively (S7 Table). These selection coefficients are well above those proposed based on population genetics studies [37, 61]. However, we did detect significant signals of polygenic adaptations using the SNPs identified in our GWAS of fertility variables. Importantly, alleles increasing survival variables or decreasing death variables were selected for (Table 3 and S8 Table). Because the alleles influencing reproductive outcomes in Tibetans are common also at low altitude, we would not expect them to have changed systematically in frequency in one subset of populations, i.e. Tibetans. Therefore, an important implication of our findings is that the alleles increasing reproductive success in Tibetans interact with either high altitude environmental conditions or with other genetic variants that are common among Tibetans but not at low altitude. This scenario strongly supports the efforts to conduct studies of genetic and phenotypic diversity in diverse populations [66] living in their ancestral environment, despite enormous logistical challenges.

An attenuated erythropoietin and Hb concentration response to hypobaric hypoxia is a hallmark phenotype of the “Tibetan pattern” of high-altitude adaptations, which is markedly different from that of Andean highlanders [32, 67, 68]. The low prevalence among Tibetans of diseases associated with elevated Hb concentration, such as chronic mountain sickness [69], and a signal of selective sweep in the *EPAS1* gene [33, 34] have led to the hypothesis that unelevated Hb is adaptive in Tibetan highlanders [70]; this hypothesis was also substantiated by the correlation between low Hb and better reproductive outcomes in our Tibetan sample [48]. Our GWAS provides the first genome-level support for the association between the Tibetan *EPAS1* haplotype and low oxyHb, which correlates highly with total Hb. Interestingly, the association was stronger for oxyHb than for total Hb (Table 2 and S4 Table), while it was not significant for deoxyHb (*p* = 0.883 for rs372272284). This observation raises the possibility that it is the oxygen-carrying portion of total Hb that drives the well-replicated association between *EPAS1* SNPs and Hb. We also found that SNPs associated with Hb did not show polygenic adaptation signals in our Tibetan sample, if the *EPAS1* SNP was excluded from the analysis (Fig 3). Intriguingly, the Sherpa, who are closely related to other Tibetan populations and also have unelevated Hb levels [41, 67, 71], show a nominally significant trend towards lower frequencies of the Hb-increasing alleles in one of the two polygenic adaptation tests (*p* = 0.019 without multiple test correction). Based on our estimate of 0.386 g/dL per allele, and a mean allele frequency difference of 0.743 between high and lowlanders, we calculated that the *EPAS1* SNPs can explain 52% of the 1.1 g/dL difference reported in [72] between Tibetan and Han Chinese women in the same age range. In our sample, the *EPAS1* SNP explains only 2.7% of inter-individual variation in Hb: therefore, almost all within-population (97.3%) as well as a substantial portion of between-population (48%) variation remains unexplained.

Several scenarios could account for these results. Incomplete power in the Tibetan GWAS and/or in the polygenic adaptation tests could underlie the lack of clear evidence for polygenic adaptation for lower Hb levels, although we had sufficient power to detect polygenic adaptation signals for several other traits in the same samples. One possibility is that post-reproductive Hb levels are a poor proxy for the levels while women are reproductively active. Some, but not all, studies of Tibetans find an increase in Hb concentration with age [73–75], but this does not imply that the relationship between genotype and phenotype also changes with age (especially if age is used as a covariate in mapping, as done here). The lack of evidence supporting low Hb as the selected trait in Tibetans stands in stark contrast with the strong selective sweep signal at *EPAS1* and with the significant evidence for polygenic adaptations toward lower deoxyHb. This finding raises the question of whether unelevated Hb was the true target of selection in Tibetans rather than a mere correlate of the true adaptive trait. This scenario would be consistent with the observed correlation between low Hb and better reproductive outcomes because pleiotropy can induce a non-causal association between phenotypes. A recent study showed that the same *EPAS1* SNP that is associated with Hb and other hematological traits is also associated with uric acid levels [38], suggesting that indeed SNPs in *EPAS1*, a transcription factor with dozens of target genes, may affect multiple, seemingly unrelated phenotypes. Interestingly, the peak of our association signal for oxyHb at *EPAS1* spans active enhancer (H3K27Ac) marks in human umbilical endothelial cells, as detected by the ENCODE project [76], pointing to gene regulatory role in the endothelium. Therefore, it could be speculated that the SNPs that influence variation in oxyHb/Hb levels also affect *EPAS1* expression in the endothelium with effects on vascularization, vasoconstriction, vasodilation and possibly beneficial effects in oxygen delivery at high altitudes. These findings suggest that the WHO altitude-adjusted elevated hemoglobin cut-off for detecting iron-deficiency anemia [77] may be inappropriate for use among Tibetan women, a result of this work that has public health implications and that warrants further research.

The lower genetic values for pulse in Tibetans compared to low altitude East Asians, coupled with the correlation between lower pulse and better reproductive outcomes in Tibetans, suggest an important role for cardiac function in pregnancy at high altitude. Intriguingly, tests of polygenic adaptation that use data from worldwide populations are consistent with selection favoring higher pulse in low altitude East Asians, but not in the closely related populations at high altitude. There is some prior evidence for selective events that took place in low altitude, but not high altitude East Asians. For example, the well-known selective sweep signal at the *ADH* locus [78] in low altitude East Asians is not shared with Tibetans: the derived allele at the nonsynonymous SNP rs3811801 is very common in Han Chinese and Japanese (CHB = 0.59 and JPT = 0.70) but relatively rare at high altitude (Tibetan = 0.08 and Sherpa = 0.07). A similar pattern is seen for the rs1800414 derived allele at the *OCA2* gene (CHB = 0.59 and JPT = 0.57 *versus* Tibetan = 0.06 and Sherpa = 0.16). Therefore, the observed shift towards higher genetic values for pulse at low altitude could be the result of a selective event that similarly affected only low altitude populations. However, the correlation between low pulse and better reproductive outcomes in Tibetans suggests that low pulse is adaptive (rather than neutral) at high altitude and raises the possibility that lower pulse was selected for when ancestral low altitude populations moved to high altitude.

Our GWAS of fertility phenotypes discovered three genome-wide significant associations (Table 2 and S1 Fig). Those signals lie in or near genes of potential biological relevance. First, the association peak for the number of pregnancies and of livebirths is located within an intron of the *CCDC141* gene (Fig 2), which is expressed in the heart and had been linked to a rare form of hypogonadotropic hypogonadism [79]. This gene is an immediate neighbor of the *TTN* (titin) gene, which codes for a major component of cardiac muscle and has been linked to idiopathic dilated and peripartum cardiomyopathy and cardiac remodeling [80, 81]. Genetic variants within 6 kb from our association peak were reported to be associated with cardiac phenotypes, such as heart rate [53, 82]. Although our GWAS signals were not associated with pulse, we hypothesize that they influence heart function, which in turn may affect pregnancy outcomes in the extreme high-altitude environments. The observed negative correlation between pulse and the number of livebirths is consistent with this idea.

Second, the top SNP in chromosome 14 associated with the number of stillbirths is 99 kb away from the *PAPOLA* gene encoding a poly-A tail polymerase that affects mRNA stability and nuclear export. Intriguingly, the product of this gene is inhibited by cordycepin, an adenosine analog (3’ deoxyadenosine), found in a fungus, “Yartsa gunbu” or *Cordyceps sinensis*, which is native to the highlands of Nepal and Tibet. Cordycepin is known to interfere with important biochemical and molecular processes, such as purine biosynthesis, DNA/RNA synthesis and mTOR (mammalian target of rapamycin) signaling transduction (reviewed in [83]). Therefore, cordycepin exposure during pregnancy could have negative effects on reproductive outcomes. Harvest of this fungus for sale primarily in China is a major source of household revenue in the Gorkha district, from where about one third of our participants were recruited. Although it is not a species consumed by ethnic Tibetan women in this region, our results raise the possibility that the *PAPOLA* SNPs may affect the stillbirth phenotype by interacting with an exposure to *C*. *sinensis* during pregnancy. An alternative and equally likely explanation is that these SNPs influence reproductive outcomes through mechanisms not involving cordycepin exposure, for example by affecting mRNA levels of key genes involved in inflammatory processes, as suggested in knockdown experiments of the *PAPOLA* gene [84], or through mechanisms involving other nearby genes.

This study was designed to extend the genetic study of human local adaptation beyond selective sweeps and candidate gene associations, by collecting genotype and physiological phenotype and reproductive history data for a large group of indigenous high-altitude Tibetan women in Nepal. Using this data set, we successfully identified several new genome-wide associations and signatures of polygenic adaptations. Our sample size of 1,000 participants is remarkably large for the genetic study of populations living in remote locations in a traditional society, but we acknowledge that is rather small for a modern-day GWAS. The census population size of ethnic Tibetans of villages in this region set the ultimate constraint on our sample size, which was obtained by recruiting virtually all inhabitants fitting our inclusion criteria. Despite this constraint, this study shows the necessity to study phenotypes of locally adapted populations in their native environments to correctly identify the adaptive phenotypes. With ever increasing throughput to generate genetic and phenotypic variation data, in-depth phenotyping of potentially adaptive features will help better understand how Tibetans and other populations living in extreme environments have adapted to their habitats.

## Materials and methods

### Ethics statement

The study protocol was approved by: the University Hospitals Institutional Review Board, University Hospitals of Cleveland (protocol no. 12-15-27), the Nepal Health Research Council, Kathmandu, Nepal (protocol no. 38/2011), the Oxford Tropical Research Ethics Committee, Oxford, UK (protocol no. 23–11), the Dartmouth College Committee for the Protection of Human Subjects (protocol no. 23374) and the Human Research Protection Office, Washington University in St. Louis (protocol no. 201202114). A written informed consent was signed by each participant.

### Sample information

A total of 1,008 ethnic Tibetan participants were recruited from high-altitude villages in Mustang and Ghorka districts in Nepal in the spring and summer of 2012. All participants were women of age 39 or older and lifelong residents above 3000 m of altitude. The study communities in Nepal lie on the southern aspects of the Tibetan Plateau. Although they are citizens of Nepal, local people speak Tibetan dialects, practice forms of religion and social organization akin to those across the Tibetan Plateau, and retain the characteristic agro-pastoral and trading mode of subsistence common among highland Tibetans [48]. An additional 103 Sherpa participants were recruited from high-altitude villages in the Khumbu district in Nepal in the summer of 2014. Most of the Sherpa participants were women of age 39 or older. We collected saliva samples of husbands and children for 12 of them. Saliva samples were collected in the field using OG-500 Oragene DNA collection kits (DNA Genotek Inc., Otawa, ON, Canada) and genomic DNA (gDNA) were extracted using the prepIT-L2P reagents (DNA Genotek Inc) following the manufacturer’s protocol. Blood hemoglobin concentration (Hb), percent arterial blood oxygen saturation (SaO_2_), and pulse rate (pulse/minute) were measured altogether using a non-invasive device Masimo Pronto-7 © (Masimo Corporation, Irvine, CA) as described in Cho et al. [48]. Two additional phenotypes, oxygenated and deoxygenated hemoglobin concentrations (oxyHb and deoxyHb, respectively), were calculated from Hb and SaO_2_ as follows: oxyHb = Hb × SaO_2_ / 100 and deoxyHb = Hb–oxyHb. For each participant, an interview session was held to retrieve detailed reproductive history as well as to collect other potential covariates. A summary of the Tibetan samples and their phenotype measurements are presented in Table 1. Detailed description of the Tibetan samples, the phenotype and covariate data collection was published in Cho et al. [48].

### Array genotyping

We generated new genome-wide genotype data for a total of 1,104 individuals indigenous to the high-altitude regions in the Himalayas in Nepal, including 1,001 ethnic Tibetans from the present study and 103 Sherpa (S1 Table). Array genotyping was performed in two phases. First, all Tibetan individuals were genotyped on 301,299 biallelic markers using the customized Illumina HumanCore-12 v1.0A array, which includes probes for additional 2,553 markers from 19 genomic loci presumed adaptive in Tibetans including the *EPAS1*, *EGLN1*, *HIF1A* and *NOS2* genes. Then, a subset of 344 unrelated Tibetans from the present study and all 103 Sherpa individuals were genotyped on 716,503 markers using the Illumina OmniExpress-24 v1.0 array to obtain denser genome-wide variation data. For each array platform, genotypes were called using the genotyping module in the Illumina Genome Studio with default parameters (GenCall score threshold 0.15). Previously defined clusters, downloadable from the Illumina website, were applied for genotype calling. For the 2,553 custom markers we added to the HumanCore array, we retrieved intensity data from the Illumina Genome Studio and performed genotype calling using the OptiCall v0.6.4 [85]. For 344 Tibetans genotyped on both Illumina platforms, we used genotype calls from the HumanCore array for the overlapping 253K markers. Genotype calls from the two platforms were highly concordant, with the average 99.98% concordance.

### Genotyping of nonsynonymous *EGLN1* SNPs

We separately genotyped two non-synonymous SNPs in the *EGLN1* gene, rs12097901 and rs188966510, in the set of 344 unrelated Tibetans. We used Epicenter FailSafe PCR system with the manufacturer’s recommended condition in buffers G and H, instead of using standard TAQ polymerases. We generated a 1,025 bp PCR fragment in an 11 ul reaction volume using a previously published primer pair PHD2-X1F (CCCCTATCTCTCTCCCCG) and PHD2-X1R (CCTGTCCAGCACAAACCC) [86]. These PCR products were sequenced using BigDye Terminator v3.1 cycle sequencing kit and the PHD2-X1F primer in an Applied Biosystems 3730XL DNA Analyzer. In a few cases where initial amplification failed, samples were diluted 4x in water, which in most cases allowed successful subsequent amplification. Genotypes were scored manually from chromatograms.

### Sample selection for whole genome sequencing

We generated novel whole genome sequence data for 18 Sherpa and 35 Tibetans from the present study, all from Nepal. Seventeen individuals were sampled with known familial relationships (four Tibetans mother-daughter duos and three Sherpa parents-offspring trios), and sequenced to high-coverage (around 20x autosomal coverages) to generate high quality phased genome sequences. The remaining 36 individuals were chosen to be unrelated and sequenced to low-coverage targeting 5x autosomal coverage.

For Sherpa, we began with 172 individuals, including 103 newly genotyped in this study and 69 previously published [41], and chose a subset of 101 unrelated individuals allowing first cousins. Coefficients of relatedness were calculated using PLINK v1.07 [87]. Then, we estimated population structure in these unrelated Sherpa, together with 30 Tibetans from near Lhasa [42] and 103 1KGP CHB, using an unsupervised genetic clustering algorithm in ADMIXTURE v1.22 [88]. Using estimates from K = 2, we chose 51 Sherpa with > 95% of their ancestry from a component enriched in Sherpa and Tibetans (the remaining portion come from an ancestry representing CHB-related low altitude East Asians). Among them, we chose three pairs of couples with their offspring and 9 additional unrelated individuals for high- and low-coverage sequencing, respectively.

For Tibetans, we ran ADMIXTURE with K = 3 in a supervised mode, with 103 1KGP CHB, 103 1KGP GIH (Gujarati Indians in Houston, Texas) and the 51 unrelated Sherpa as three reference groups. Pairwise relatedness was then calculated with the ADMIXTURE output using the RelateAdmix v0.08, controlling for population structure due to admixture [89]. Among individuals with minimum South Asian ancestry (< 1%), represented by GIH, we chose four pairs of mother-daughter duos of Tibetans from the present study and 27 unrelated individuals for high- and low-coverage sequencing, respectively.

### Whole genome sequencing

Single-barcoded libraries for Illumina sequencing were constructed using the TruSeq library preparation kit. Libraries were pooled into multiple batches and sequenced in the Illumina HiSeq 2500 and 4000 machines for paired-end (PE) 100 and 125 bp designs (S1 Table). Sequence reads were demultiplexed with no mismatch in 6-bp barcode sequence allowed. Reads were mapped to the human reference genome sequences (hg19) downloaded from http://hgdownload.soe.ucsc.edu/goldenPath/hg19/chromosomes/, using BWA backtrack v0.7.4 with -q15 option [90]. PCR and optical duplicate reads were marked using Picard tools v1.98 (http://broadinstitute.github.io/picard/) and were excluded from further analysis. Local realignment around indels and base quality score recalibration were performed using the GenomeAnalysis ToolKit (GATK) v2.8–1, following the best practice pipeline [91–93]. Finally, analysis-ready BAM files for variant discovery and genotype calling were produced using Samtools v1.2 [94] by filtering out reads with Phred-scaled mapping quality lower than 30.

LD-aware variant and genotype calling was performed using the GotCloud pipeline [95] with default parameters. The analysis-ready BAM files of 53 newly sequenced individuals and 6 previously reported ones, four Sherpa and two Nepali Tibetans [41, 43], were provided to the pipeline together.

### Imputation of array genotype data

We performed genotype imputation of Tibetan and Sherpa samples, which were array-genotyped either in the present or in our previous study [41] (S1 Table). For each array genotyping platform, low quality markers and samples were filtered out by applying the following filters: per-marker missing rate ≤ 0.05, Hardy-Weinberg equilibrium (HWE) *p*-value ≥ 0.00001 and per-individual missing rate ≤ 0.03. Strand-ambiguous (A/T and G/C) SNPs were removed and only SNPs in autosomes or X chromosome were retained for imputation. The filtering process was performed using PLINK v1.90 [96]. Genotype imputation was performed for each set of samples separately using IMPUTE2 v2.3.2 [46]. We used both our phased genotype calls of 59 high-altitude samples and the 1KGP phase 3 reference data set, downloadable from https://mathgen.stats.ox.ac.uk/impute/1000GP_Phase3.html, as imputation references by merging them with “-merge_ref_panels” flag in IMPUTE2. For other parameters, we used default values set by the program. Following imputation, genotypes with posterior probability ≥ 0.9 were accepted. Genotypes were assumed to be missing if none of three possible genotypes reached posterior probability threshold of 0.9. Then, we conducted an additional round of quality control by removing SNPs with missing rate higher than 0.05 or HWE *p*-value smaller than 10^−6^.

### Genome-wide association analysis

Among 1,001 successfully genotyped and imputed Tibetan women, 991 individuals were included in our genome-wide association analysis (GWAS). Four individuals were excluded from the analysis because they were born below 3,000 m. Another individual was excluded from the analysis was a genetic outlier who clustered with individuals from the Indian subcontinent. The other five were excluded either because they had inconsistent reproductive record or because they were nuns who became celibate during their reproductive years.

For physiological phenotypes, we chose relevant covariates by performing a stepwise model selection, allowing removal of a single covariate each step if likelihood ratio test (LRT) *p*-value obtained from the “lrtest” function in the R “lmtest” library was bigger than 0.05. The final sets of chosen covariates for physiological covariates are listed in S3 Table. For fertility phenotypes, we used an *a priori* chosen set of four covariates: age, subdistrict, use of contraception and “continuously married (CM)” status. Use of contraception was categorized into three classes: never used, previously used, and currently in use. “Continuously married” status is a binary variable defined as being in a marital relationship throughout the ages of 25 and 40. It includes two who had experienced less than two years of gap before re-marriage following divorce or death of the husband. Table 1 presents a summary of these covariates. A full list of GWAS phenotypes and their description are provided in S3 Table.

GWAS was performed using GEMMA v0.94.1 [47]. Univariate linear mixed model (LMM) as implemented in GEMMA was used to control for both population structure and genetic relatedness [47]. For each phenotype, we first removed individuals with no information on either the focal phenotype or its associated covariates. Second, we kept SNPs with maf ≥ 5% for the chosen subset of individuals. Third, the standardized genetic covariance matrix was calculated from this data set and was used for LMM. Last, GWAS was run controlling for the above covariates. For continuous and count data, we provided raw phenotype data together with covariates to the program. For the binomial data, we fitted a binomial regression model using the “glm” function in R, calculated the difference between the observed odds and the odds of the fitted value, and used this residual as a GWAS phenotype. LRT *p*-values from GEMMA were used to assess significance of genetic association. *P*-values of the full and subsample sets were highly correlated for each fertility phenotype (Pearson *r* = 0.36–0.74 with *p* < 10^−15^ for–log_10_ transformed *p*-values).

### Polygenic adaptation signals

To detect signatures of polygenic adaptation, we investigated systematic changes in allele frequencies of SNPs associated with each phenotype. For all of Tibetan GWAS phenotypes, we first took all SNPs with *p* ≤ 10^−4^ and lumped them into peaks by allowing maximum inter-SNP distance of 200 kb. Finally, we chose one SNP with the smallest association *p*-value for each peak to retrieve a set of independently associated SNPs. We also retrieved a set of SNPs associated with blood hemoglobin level (Hb) using summary statistics from a published large-scale GWAS meta-analysis [58]. To obtain a list of independent markers, we first confined markers to those overlapping with our Tibetan data and applied a more stringent cutoff of *p* ≤ 10^−5^. Then, we removed SNPs in LD: for each pair of SNPs with r^2^ > 0.2 in 1KGP CEU, we removed one with larger association *p*-value.

After retrieving phenotype-associated SNPs with their effect size, we first compared mean frequency difference of trait-increasing alleles between Tibetans and 1KGP CHB. Following [13], we sampled 10,000 sets of random SNPs, where each set contained an equal number of SNPs as the GWAS SNPs matched one-to-one by mean minor allele frequency in bins of size 0.02. The empirical distribution of mean frequency difference of trait-increasing alleles was compared to the observed value from the GWAS SNPs and the empirical one-sided *p*-value was calculated as the proportion of random SNP sets with their mean allele frequency difference equal to or more extreme than the observed one.

We also looked into comprehensive signatures of polygenic adaptation using a machinery introduced by [14]. It requires as an input a set of SNPs associated with the target phenotype together with their allele frequency and effect size estimate. For each population, a “genetic value” of the target phenotype is calculated as a weighted sum of population allele frequency over the GWAS SNPs with the effect size as a weight. Then, the calculated genetic value is used for a set of tests asking i) if the GWAS SNPs are collectively more differentiated between populations than the matched random SNPs are, ii) if the direction of allele frequency differentiation is more consistent in GWAS SNPs than in matched random SNPs, iii) if the genetic value is correlated with an environmental variable over populations, or iv) if a regional group or a population’s genetic value is away from the expected value by the genetic value of the other populations. For this, we used allele frequency of 26 populations in the 1KGP phase 3 data set overlapping with the Tibetan data. We first sampled random SNPs matching each of the GWAS SNPs by minor allele frequency bin of size 0.02 in the GWAS population and by the B-value bin of size 100 (values ranging from 0 to 1,000) [97]. We sampled up to several thousands of random SNPs per GWAS SNP to obtain around 100,000 random SNPs in total. These random SNPs were used for calculating the genetic covariance matrix of populations and for generating 5,000 sets of matched random SNPs.

### Connecting selection coefficient and the statistical power to detect genotype-phenotype association

To estimate the strength of positive selection necessary to generate a significant association between the fertility count phenotypes and genotype in our sample of the unrelateds, we assumed a simple additive model. That is, genotypes with 0, 1 and 2 adaptive alleles, with population frequency *p*, have the mean absolute fitness *W*_*0*_, *W*_*0*_ (1+s) and *W*_*0*_ (1+2s). Using the observed mean phenotype value, *W*_*m*_, we can get the per-allele effect size s*W*_*0*_ as a function of s, *W*_*m*_ and *p*:

$$sW_{0}=\frac{sW_{m}}{1+2sp}$$

Then, the effect size was standardized to the unit of standard deviation, using the observed standard deviation of the phenotype. For the standardized effect size, which is a function of selection coefficient *s*, the statistical power to detect association was calculated using the “pwr.r.test” function in the R package “pwr”.

## Supporting information

S1 Fig GWAS quantile-quantile (QQ) and Manhattan plots for fertility phenotypes with genome-wide significant associations: (A, B) the number of pregnancies, (C, D) the number of live births, (E, F) the number of stillbirths, (G-J) the proportion of children born alive but died < 15 yr. (E, F, I, J) show GWAS results using the continuously married (“CM”) subset of individuals.
(TIF)

S2 Fig GWAS quantile-quantile (QQ) and Manhattan plots for five physiological phenotypes in Tibetans: Hb (A, B), SaO_2_ (C, D), Pulse (E, F), oxyHb (G, H) and deoxyHb (I, J).
(TIF)

S3 Fig Locuszoom plots of the conditional association between markers around the *EPAS1* gene and (A) Hb and (B) oxyHb, conditional on the genotypes of the top *EPAS1* SNP rs372272284. No residual association was observed.
(TIF)

S4 Fig Tests of polygenic adaptation of oxyHb- and deoxyHb-associated SNPs in Tibetans: (A, B) all 43 oxyHb-associated SNPs (*p* ≤ 10^−4^), (C, D) 42 oxyHb-associated SNPs after excluding the *EPAS1* SNP rs372272284, and (E, F) 45 deoxyHb-associated SNPs (*p* ≤ 10^−4^). (A, C, E) The mean frequency difference of trait-increasing alleles was presented (solid red line) together with the empirical null distribution of 10,000 sets of matched random SNPs. (B, D, F) The genetic values of populations (filled dots) and of regions (horizontal lines) were plotted. The size of dots and the width of lines are proportional to the significance of the corresponding outlier test.
(TIF)

S5 Fig Tests of polygenic adaptation of pulse-associated SNPs (A, B) in Tibetans (*p* ≤ 10^−4^; n = 52) or (C, D) in the UK Biobank data (*p* ≤ 10^−9^; n = 123). (A, C) The mean frequency difference of trait-increasing alleles was presented (solid red line) together with the empirical null distribution of 10,000 sets of matched random SNPs. (B, D) The genetic values of populations (filled dots) and of regions (horizontal lines) were plotted. The size of dots and the width of lines are proportional to the significance of the corresponding outlier test.
(TIF)

S6 Fig Tests of polygenic adaptation of six fertility phenotypes reaching nominal significance (*p* < 0.05) for both the mean frequency difference and the outlier tests: (A, B) the number of children surviving at 1 yr, (C, D) the number of live births, (E, F) the number of children born alive but died < 1 yr, (G, H) the number of children born alive but died < 15 yr, (I, J) the number of miscarriages, and (K, L) the number of twin births. (C-L) show GWAS results using the continuously married (“CM”) subset, while (A, B) show GWAS results using all individuals.
(TIF)

S7 Fig Principal component analysis of Eurasian populations. The first two principal components (PCs) calculated from 1,295 individuals belonging to 132 groups (S9 Table) are plotted. Each three code represents a single individual, here colored by geographic regions. Ethnic Tibetan women from the Himalayan valleys, marked by color-filled symbols, are not included in calculating PC to avoid distortion due to their large sample size. Instead, they are projected onto calculated PCs using “*lsqproject*: *YES*” option. Numbers in the parenthesis in the axis label show the percentage of total genetic variation explained by each PC.
(TIF)

S8 Fig Principal component analysis of East Asian populations. The first two principal components (PCs) calculated from 357 individuals belonging to 31 groups (S9 Table) are plotted. Each three code represents a single individual, here colored by geographic regions. Ethnic Tibetan women from the Himalayan valleys, marked by color-filled symbols, are not included in calculating PC to avoid distortion due to their large sample size. Instead, they are projected onto calculated PCs using “*lsqproject*: *YES*” option. Numbers in the parenthesis in the axis label show the percentage of total genetic variation explained by each PC.
(TIF)

S9 Fig The genetic affinity of the Himalayan Tibetan groups with world-wide populations. (A-D) The top 15 outgroup-*f*_*3*_ signals, measuring shared genetic drift from a common outgroup Mbuti, are plotted for (A) Nubri, (B) Tsum, (C) Lower Mustang and (D) Upper Mustang. (E-F) The extra genetic affinity with South Asians (Brahui, Kalash, Pathan) of the Himalayan Tibetans compared to (E) Tibetans from Lhasa and (F) Sherpas are measured by *f*_*4*_ statistics. Thick and thin horizontal bars represent ± 1 and 3 standard errors, respectively, estimated by 5 cM block jackknifing.
(TIF)

S10 Fig South Asian ancestry proportion of the Himalayan Tibetan individuals. The distribution of (A) altitude of residence and (B) South Asian ancestry proportion across individuals are summarized into a violin plot, using R package “vioplot”. South Asian ancestry proportion is estimated by fitting a two-way admixture model of Sherpa+Pathan using the qpAdm program. In panel (B), grey triangles show South Asian ancestry proportion estimated by group. Group-based estimates match well with the mean value of individual-based estimates.
(TIF)

S11 Fig A negative correlation between altitude of residence and South Asian ancestry proportions. Each color-filled symbols represents a single individual. The red solid line shows a simple linear regression line. The observed negative correlation is highly significant even after controlling for the sub-district label (effect size *β* = -1.835×10^−5^, *p* = 2.89×10^−9^).
(TIF)

S12 Fig Admixture date estimates of the Himalayan Tibetans. The solid line curves show an exponential fit to the observed weighted admixture LD decay using ALDER. Point estimates ± one standard error estimates are shown in each panel. Standard errors are estimated by leave-one-chromosome-out approach, as implemented in ALDER.
(TIF)

S1 Table Indigenous high-altitude individuals for whole genome sequencing and Illumina array genotyping. (PDF)

S2 Table A summary of multi-sample variant calling of 59 high-altitude genomes. (PDF)

S3 Table Phenotypes for genome-wide association analysis. (PDF)

S4 Table The eight *EPAS1* SNPs on chromosome 2 with genome-wide significant association with oxyHb. POS column is for the genomic position in hg19. REF, ALT and DEN columns show reference, alternative and Denisovan alleles, respectively. TBN and CHB present alternative allele frequencies in our Tibetan data and 1KGP CHB, respectively. Effect size estimates *β*’s are calculated per alternative allele. SNPs with a derived allele shared between Tibetans and Denisovan are marked in bold face.
(PDF)

S5 Table The 189 genomic regions harboring excess top 0.1% PBS SNPs in Tibetans. (XLSX)

S6 Table *P*-values for the correlation between fertility and physiological phenotypes. Tests with *p* ≤ 0.01 were marked with grey shades. Positive regression coefficients are marked in red color.
(PDF)

S7 Table *P* values for the correlation between fertility or fertility proportion phenotypes controlling for covariates and the *EGLN1* and *EPAS1* SNP genotypes. Per-allele selection coefficient for 80% power to detect association in a single SNP test (α = 0.05) was estimated for fertility count phenotypes. No test showed *p* ≤ 0.01.
(PDF)

S8 Table Results of polygenic adaptation tests for the GWAS phenotypes. (PDF)

S9 Table A list of 240 groups used for the population genetic analyses in this study. This list includes 182 present-day and 58 ancient groups. “N” column shows the total number of individuals in each group. Four sub-district groups of the Himalayan Tibetans reported in this study are marked by yellow color. For each group, we assigned a unique three-letter code (“Symbol”) and used it across figures. “Eurasian” and “East Asian” PCA columns mark populations used for calculating PCs for each set. “F-statistics” column marks populations used for calculating world-wide outgroup-*f*_*3*_ and *f*_*4*_ statistics. For each group, we show in which studies samples belonging to it were published in the “Study” column.
(XLSX)

S10 Table Two-way admixture modeling of the Himalayan Tibetans using the qpAdm program. Sherpas from the Khumbu region and a South Asian population are used as sources. All four sub-district groups are adequately approximated by the two-way model (*P*_*2way*_ ≥ 0.05). Coef_1_, Coef_2_ and SE columns represent estimated ancestry proportions from Ref_1_ (Sherpa) and Ref_2_ (South Asian), as well as their associated standard errors. Although the South Asian ancestry proportion is small (2.5–6.2%), it is necessary to explain the Himalayan Tibetans, as shown by the insufficiency of the Sherpa-only model (*P*_*1way*_ << 0.05).
(PDF)

S11 Table Estimates of power to detect an association between the *EGLN1* SNP rs186996510 and Hb in our data set. We used effect size estimates from multiple studies, ranging from 0.386 to 1.676 g/dL per allele. Power was calculated using the observed allele frequency (maf = 0.336), sample size (n = 649) and standard deviation of Hb residuals after regressing out covariates (1.330 g/dL), assuming a single test (α = 0.05).
(PDF)

S12 Table Correlation of GWAS–log_10_(*p*) between the main GWAS run including relatives and the one including unrelated individuals only. For each phenotype, we present the number of individuals included in the main GWAS (n_ind_), the number of unrelated individuals (n_unrelated_), and Pearson’s correlation coefficient across overlapping SNPs (*r*). For fertility phenotypes, we present results both for all-sample set and for the continuously married subset.
(PDF)

S1 Text Limited South Asian admixture in ethnic Tibetans from the Himalayan valleys in Nepal. (PDF)

S2 Text No genetic association between the nonsynonymous *EGLN1* SNPs and Hb phenotypes. (PDF)

S3 Text Genomic signatures of recent selective sweeps in Tibetans. (PDF)
