# Supplementary figures and images for: Detecting past and ongoing natural selection among ethnically Tibetan women at high altitude in Nepal
Source: PLoS Genet. 2018 Sep 6;14(9):e1007650. doi: 10.1371/journal.pgen.1007650 (PMC6143271; doi:10.1371/journal.pgen.1007650)

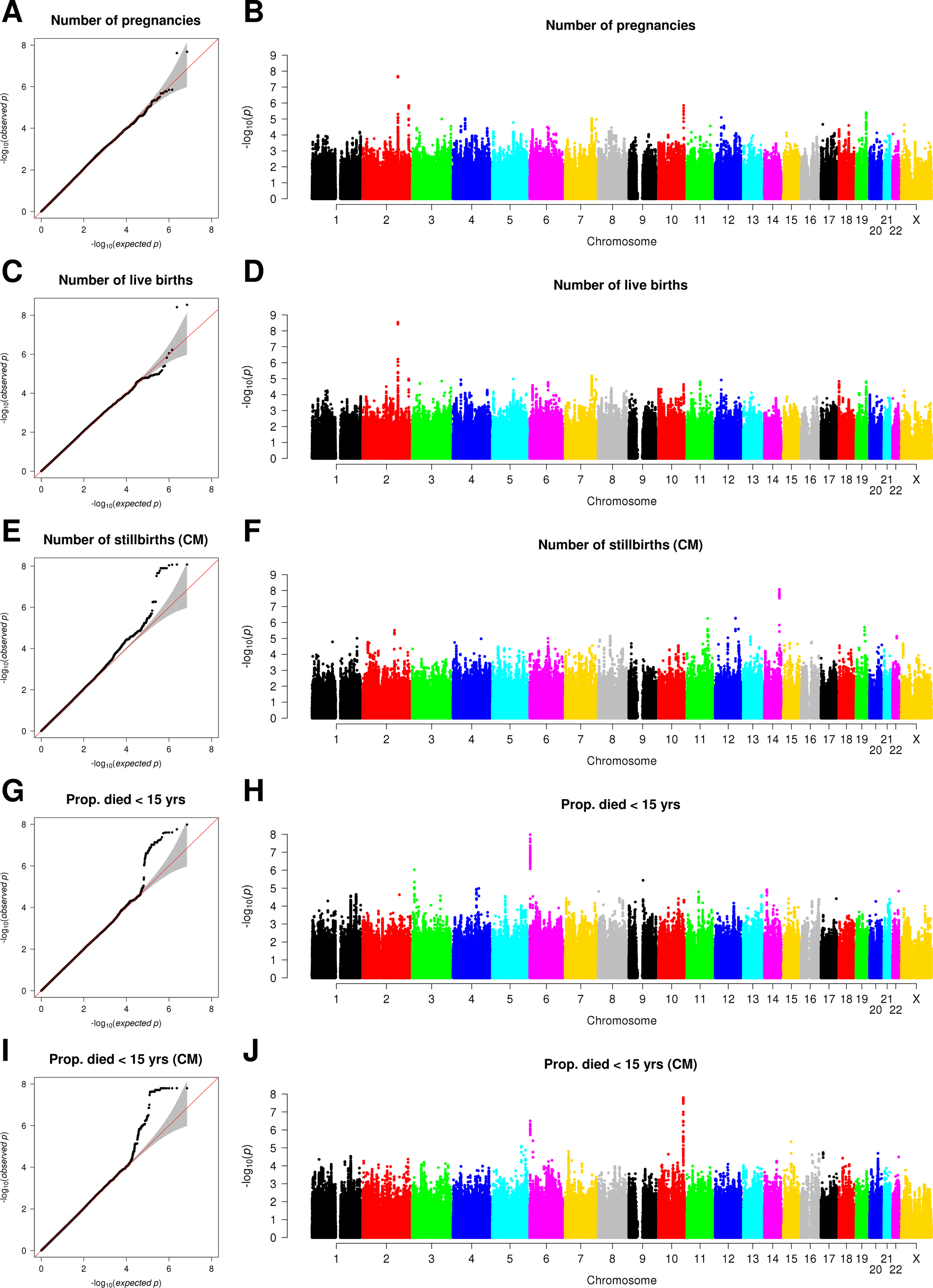

Supplement: S1 Fig — GWAS quantile-quantile (QQ) and Manhattan plots for fertility phenotypes with genome-wide significant associations: (A, B) the number of pregnancies, (C, D) the number of live births, (E, F) the number of stillbirths, (G-J) the proportion of children born alive but died < 15 yr. (E, F, I, J) show GWAS results using the continuously married (“CM”) subset of individuals. (TIF) [file pgen.1007650.s001.tif]

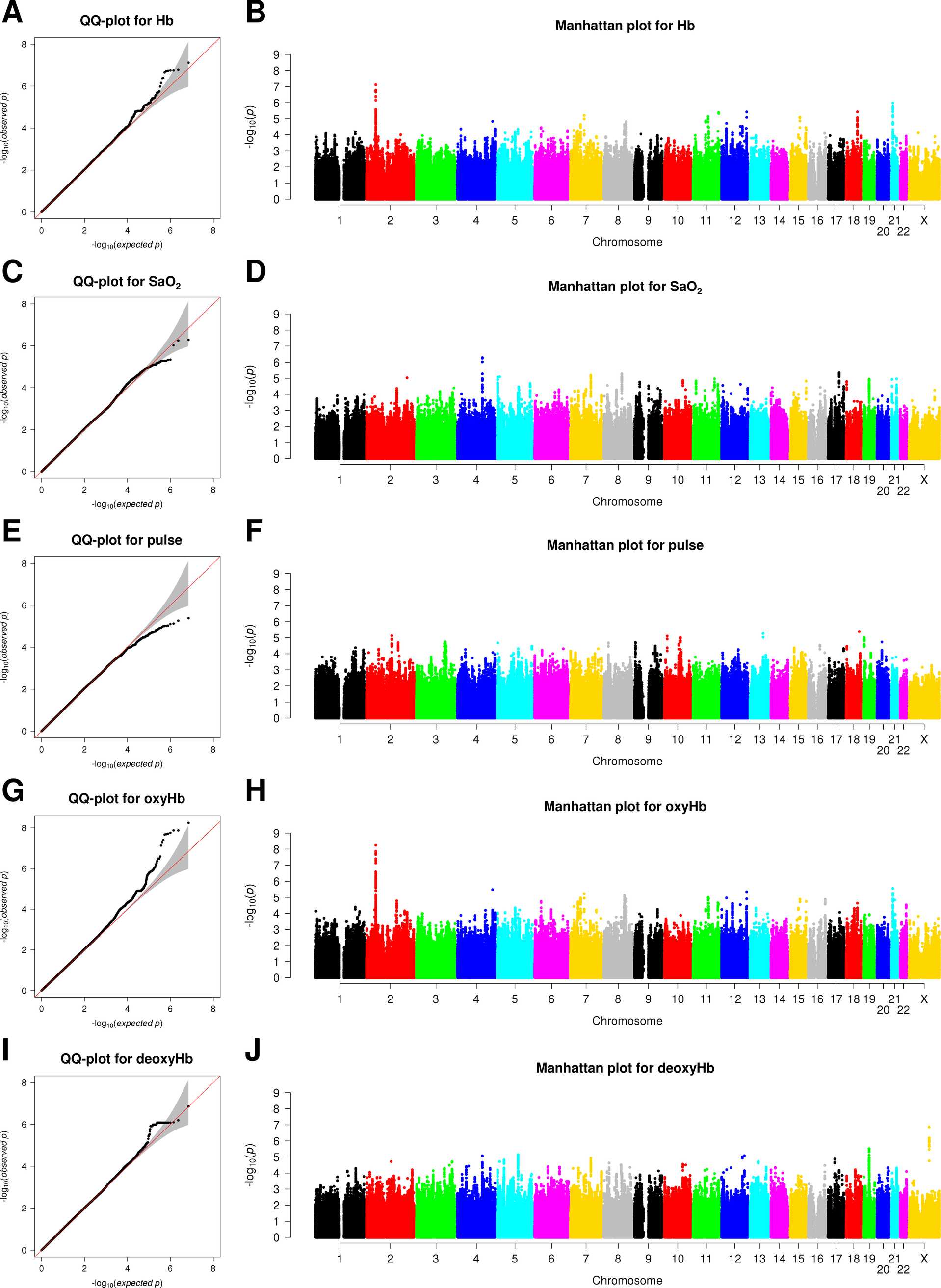

Supplement: S2 Fig — GWAS quantile-quantile (QQ) and Manhattan plots for five physiological phenotypes in Tibetans: Hb (A, B), SaO2 (C, D), Pulse (E, F), oxyHb (G, H) and deoxyHb (I, J). (TIF) [file pgen.1007650.s002.tif]

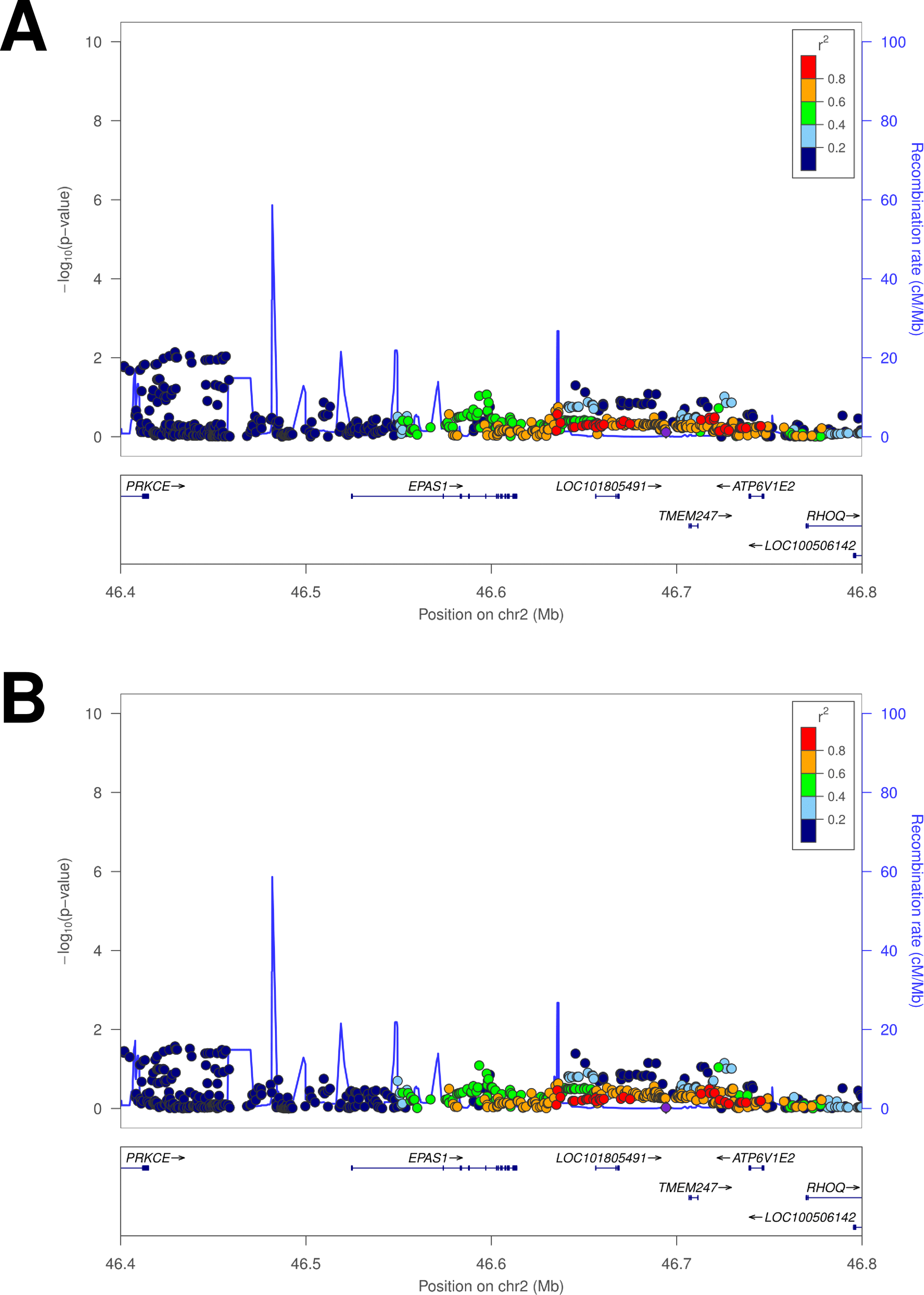

Supplement: S3 Fig — Locuszoom plots of the conditional association between markers around the EPAS1 gene and (A) Hb and (B) oxyHb, conditional on the genotypes of the top EPAS1 SNP rs372272284. No residual association was observed. (TIF) [file pgen.1007650.s003.tif]

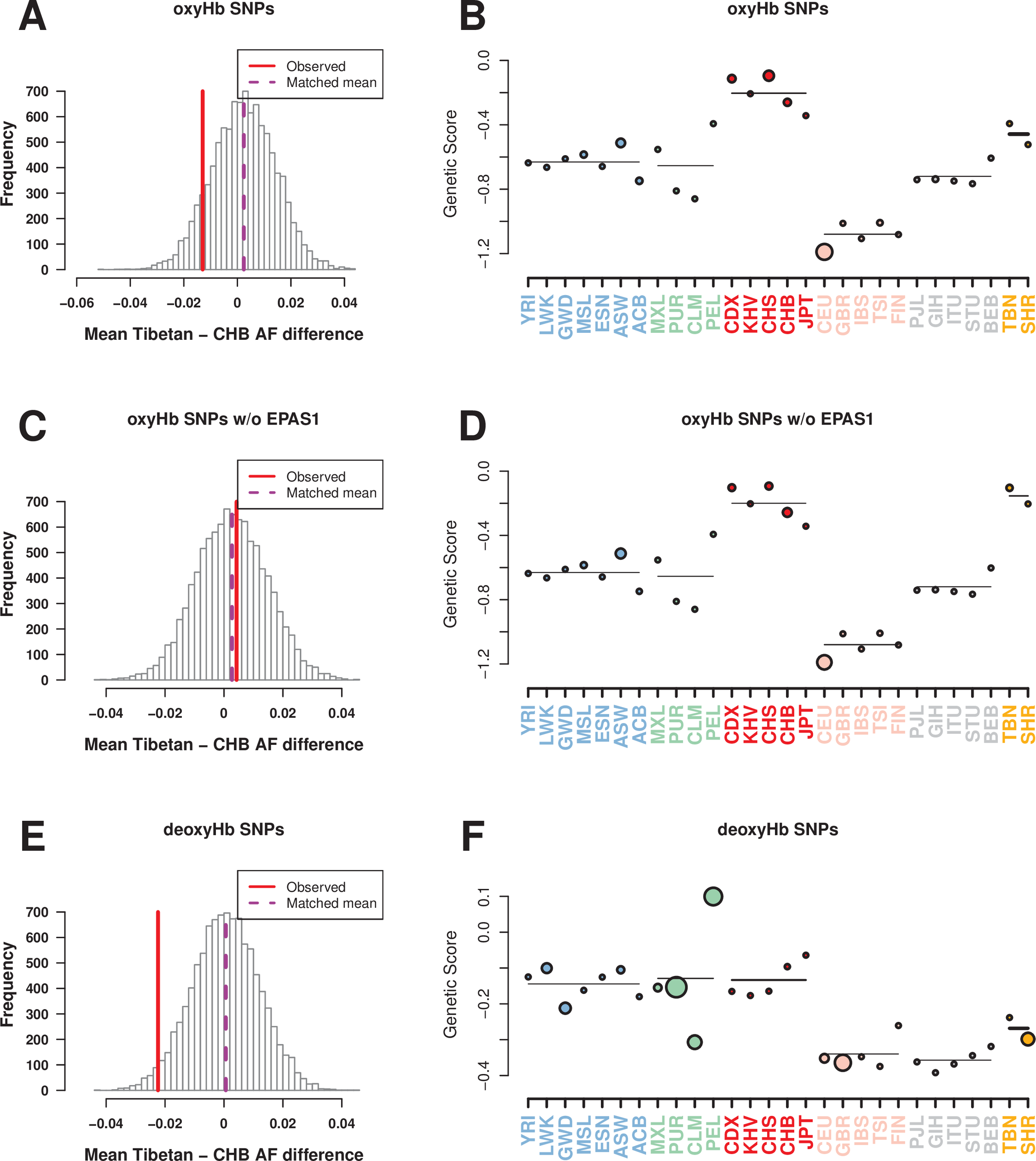

Supplement: S4 Fig — Tests of polygenic adaptation of oxyHb- and deoxyHb-associated SNPs in Tibetans: (A, B) all 43 oxyHb-associated SNPs (p ≤ 10−4), (C, D) 42 oxyHb-associated SNPs after excluding the EPAS1 SNP rs372272284, and (E, F) 45 deoxyHb-associated SNPs (p ≤ 10−4). (A, C, E) The mean frequency difference of trait-increasing alleles was presented (solid red line) together with the empirical null distribution of 10,000 sets of matched random SNPs. (B, D, F) The genetic values of populations (filled dots) and of regions (horizontal lines) were plotted. The size of dots and the width of lines are proportional to the significance of the corresponding outlier test. (TIF) [file pgen.1007650.s004.tif]

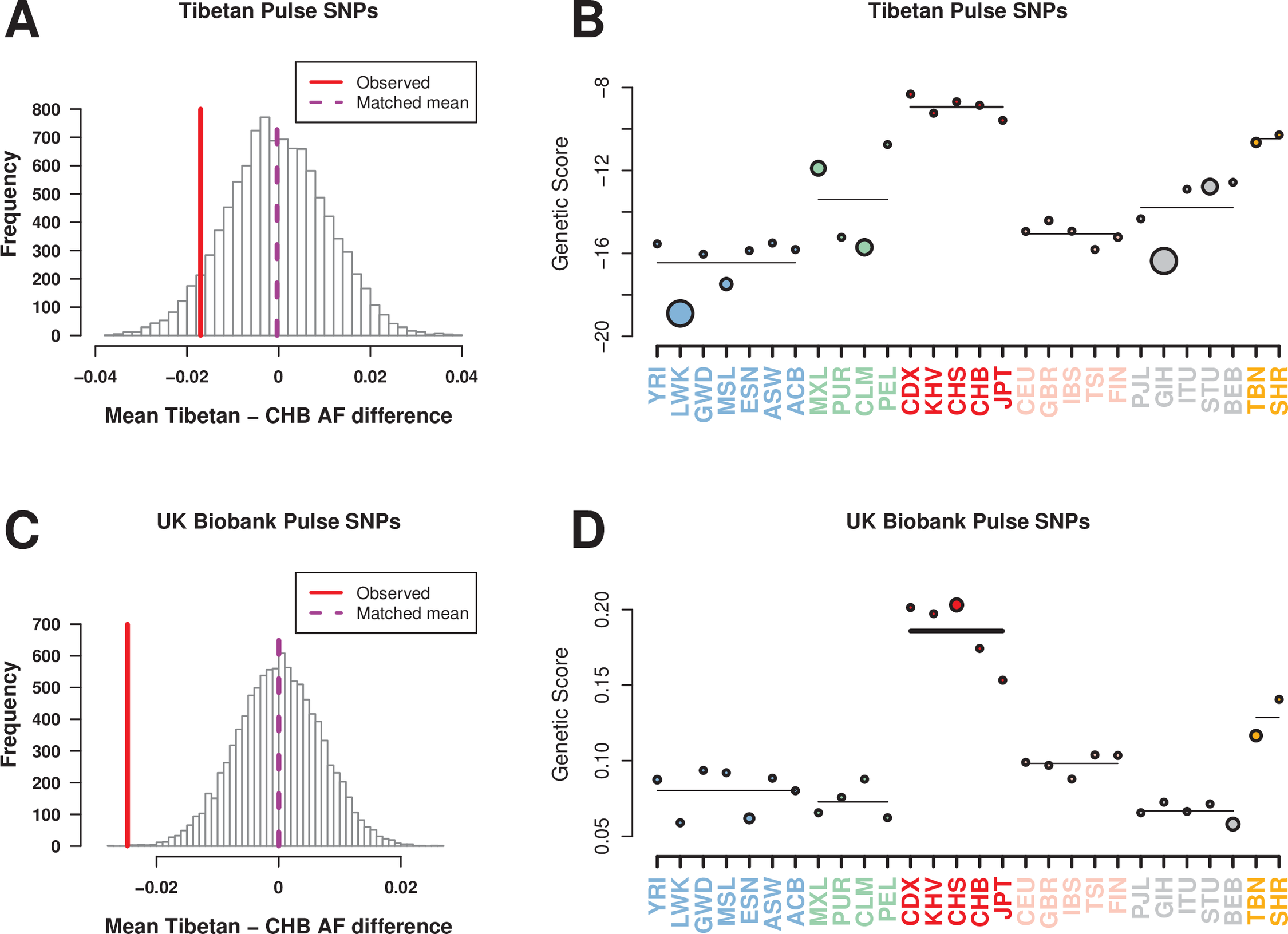

Supplement: S5 Fig — Tests of polygenic adaptation of pulse-associated SNPs (A, B) in Tibetans (p ≤ 10−4; n = 52) or (C, D) in the UK Biobank data (p ≤ 10−9; n = 123). (A, C) The mean frequency difference of trait-increasing alleles was presented (solid red line) together with the empirical null distribution of 10,000 sets of matched random SNPs. (B, D) The genetic values of populations (filled dots) and of regions (horizontal lines) were plotted. The size of dots and the width of lines are proportional to the significance of the corresponding outlier test. (TIF) [file pgen.1007650.s005.tif]

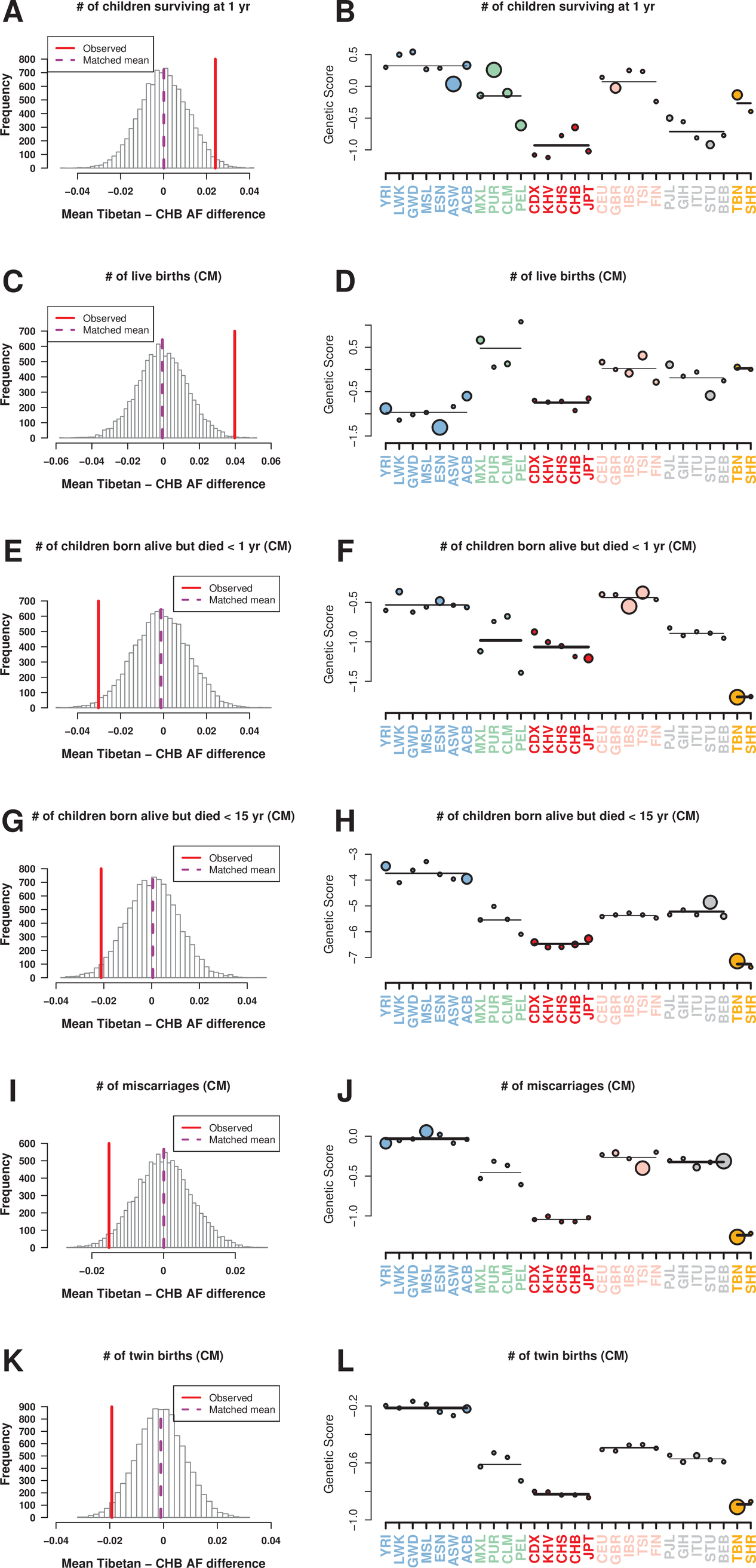

Supplement: S6 Fig — Tests of polygenic adaptation of six fertility phenotypes reaching nominal significance (p < 0.05) for both the mean frequency difference and the outlier tests: (A, B) the number of children surviving at 1 yr, (C, D) the number of live births, (E, F) the number of children born alive but died < 1 yr, (G, H) the number of children born alive but died < 15 yr, (I, J) the number of miscarriages, and (K, L) the number of twin births. (C-L) show GWAS results using the continuously married (“CM”) subset, while (A, B) show GWAS results using all individuals. (TIF) [file pgen.1007650.s006.tif]

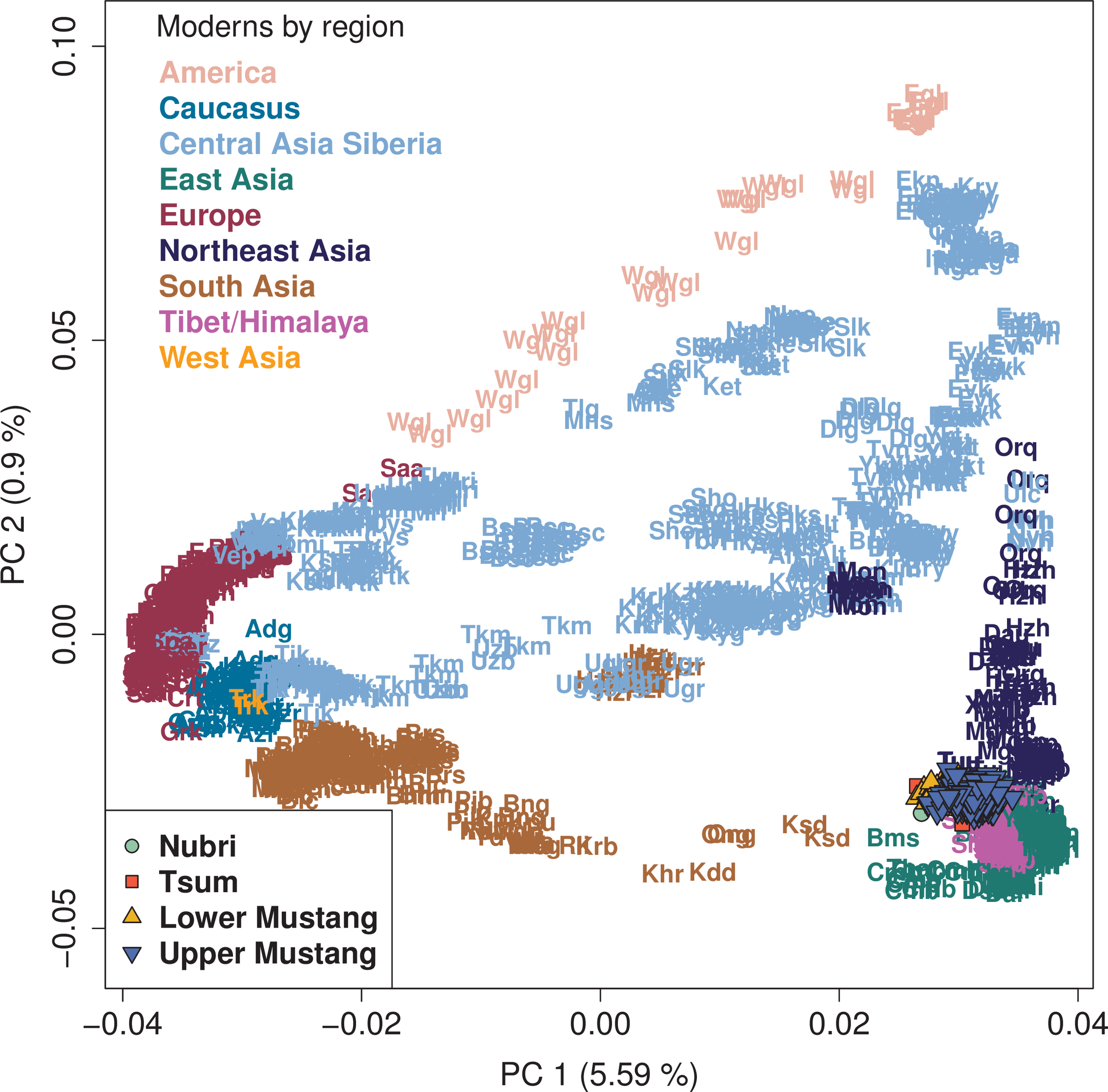

Supplement: S7 Fig — The first two principal components (PCs) calculated from 1,295 individuals belonging to 132 groups (S9 Table) are plotted. Each three code represents a single individual, here colored by geographic regions. Ethnic Tibetan women from the Himalayan valleys, marked by color-filled symbols, are not included in calculating PC to avoid distortion due to their large sample size. Instead, they are projected onto calculated PCs using “lsqproject: YES” option. Numbers in the parenthesis in the axis label show the percentage of total genetic variation explained by each PC. (TIF) [file pgen.1007650.s007.tif]

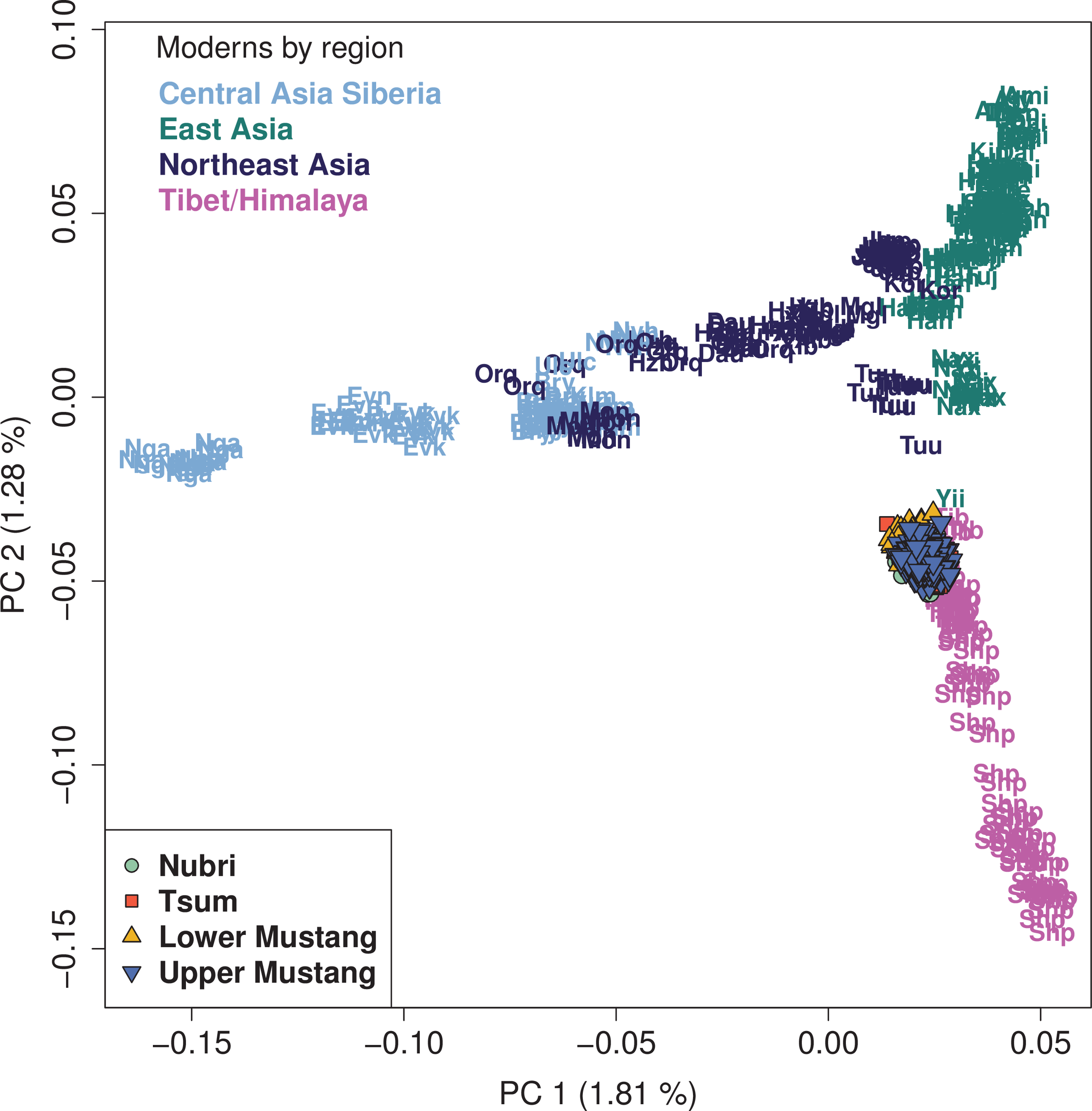

Supplement: S8 Fig — The first two principal components (PCs) calculated from 357 individuals belonging to 31 groups (S9 Table) are plotted. Each three code represents a single individual, here colored by geographic regions. Ethnic Tibetan women from the Himalayan valleys, marked by color-filled symbols, are not included in calculating PC to avoid distortion due to their large sample size. Instead, they are projected onto calculated PCs using “lsqproject: YES” option. Numbers in the parenthesis in the axis label show the percentage of total genetic variation explained by each PC. (TIF) [file pgen.1007650.s008.tif]

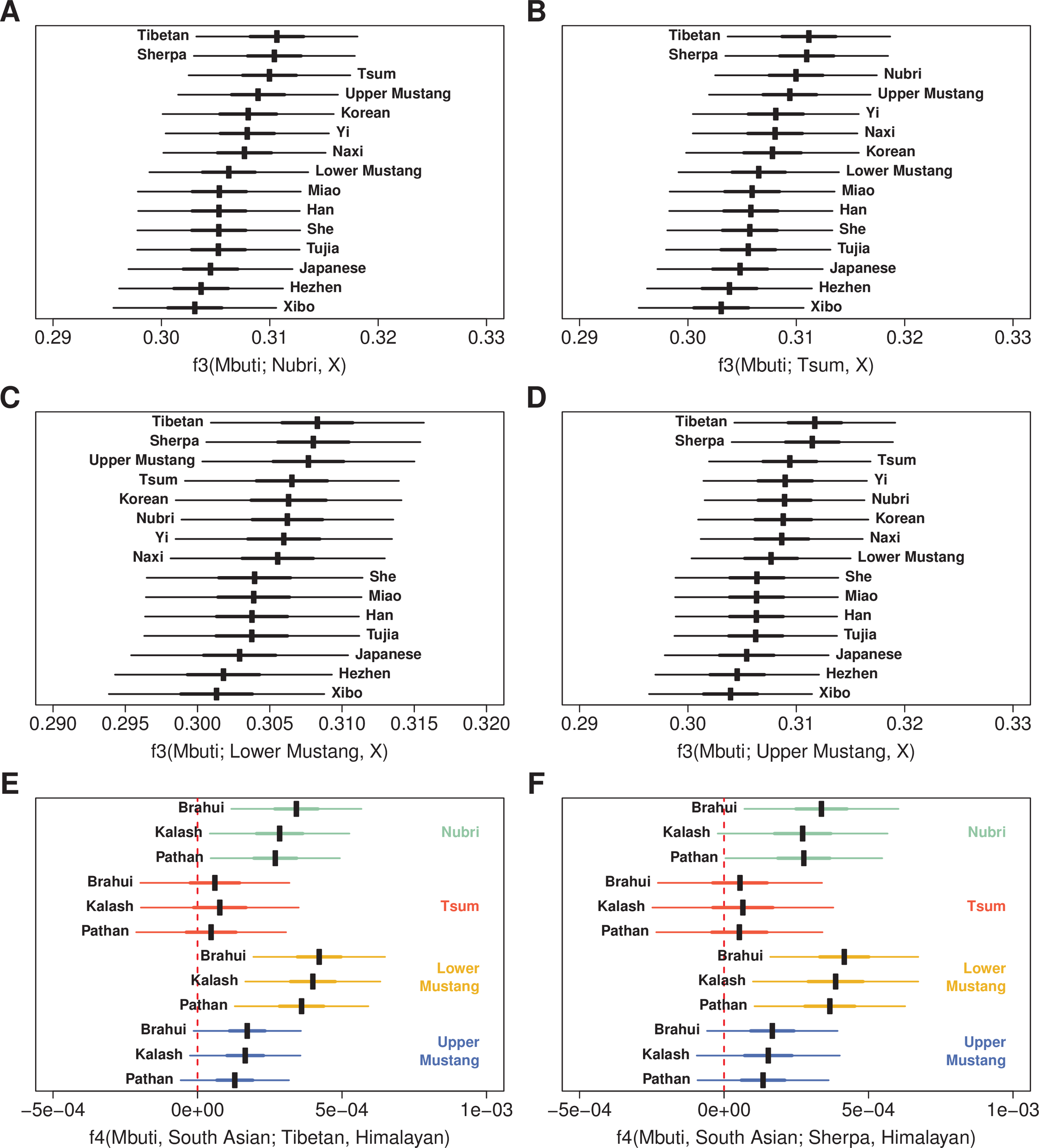

Supplement: S9 Fig — (A-D) The top 15 outgroup-f3 signals, measuring shared genetic drift from a common outgroup Mbuti, are plotted for (A) Nubri, (B) Tsum, (C) Lower Mustang and (D) Upper Mustang. (E-F) The extra genetic affinity with South Asians (Brahui, Kalash, Pathan) of the Himalayan Tibetans compared to (E) Tibetans from Lhasa and (F) Sherpas are measured by f4 statistics. Thick and thin horizontal bars represent ± 1 and 3 standard errors, respectively, estimated by 5 cM block jackknifing. (TIF) [file pgen.1007650.s009.tif]

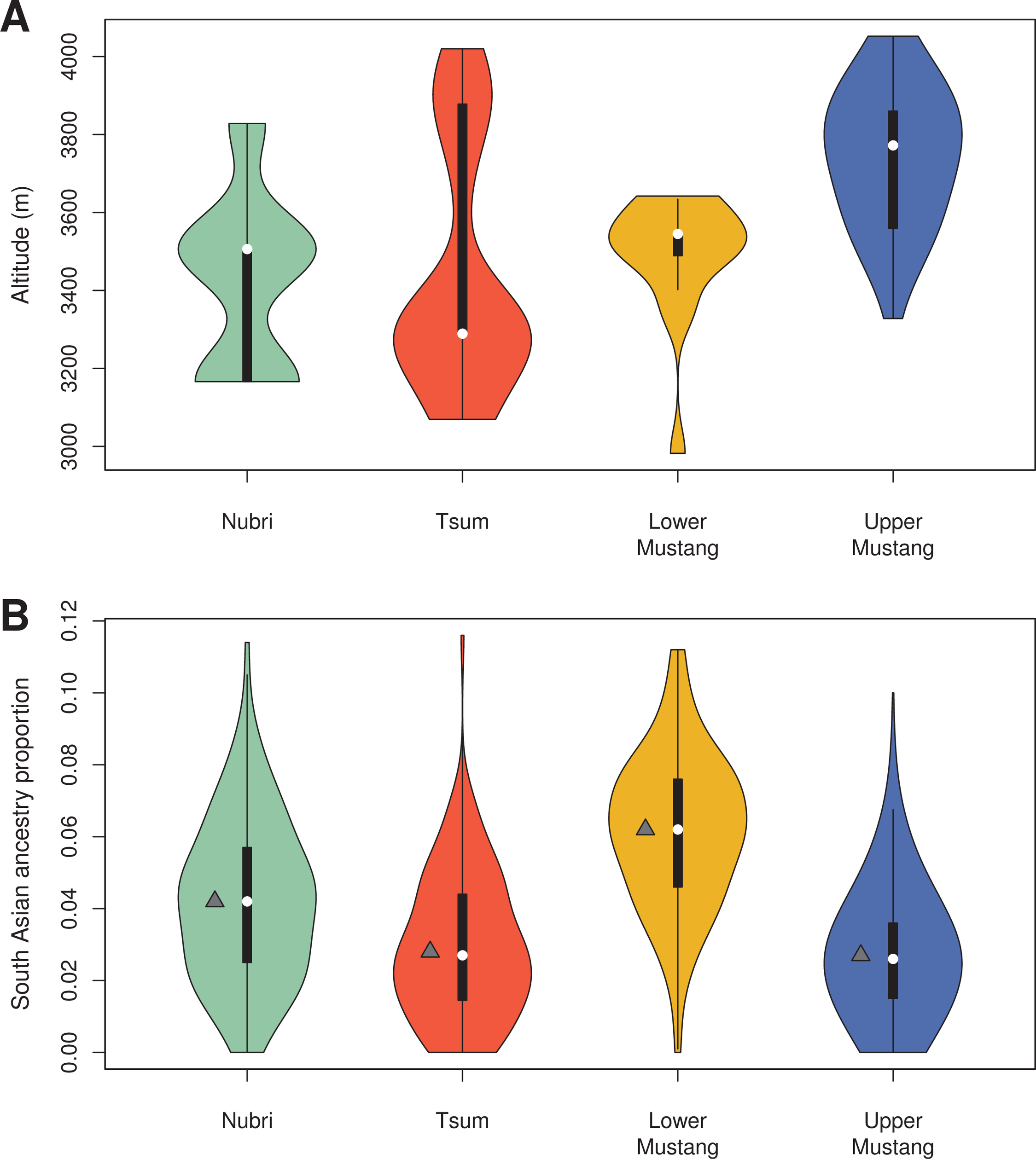

Supplement: S10 Fig — The distribution of (A) altitude of residence and (B) South Asian ancestry proportion across individuals are summarized into a violin plot, using R package “vioplot”. South Asian ancestry proportion is estimated by fitting a two-way admixture model of Sherpa+Pathan using the qpAdm program. In panel (B), grey triangles show South Asian ancestry proportion estimated by group. Group-based estimates match well with the mean value of individual-based estimates. (TIF) [file pgen.1007650.s010.tif]

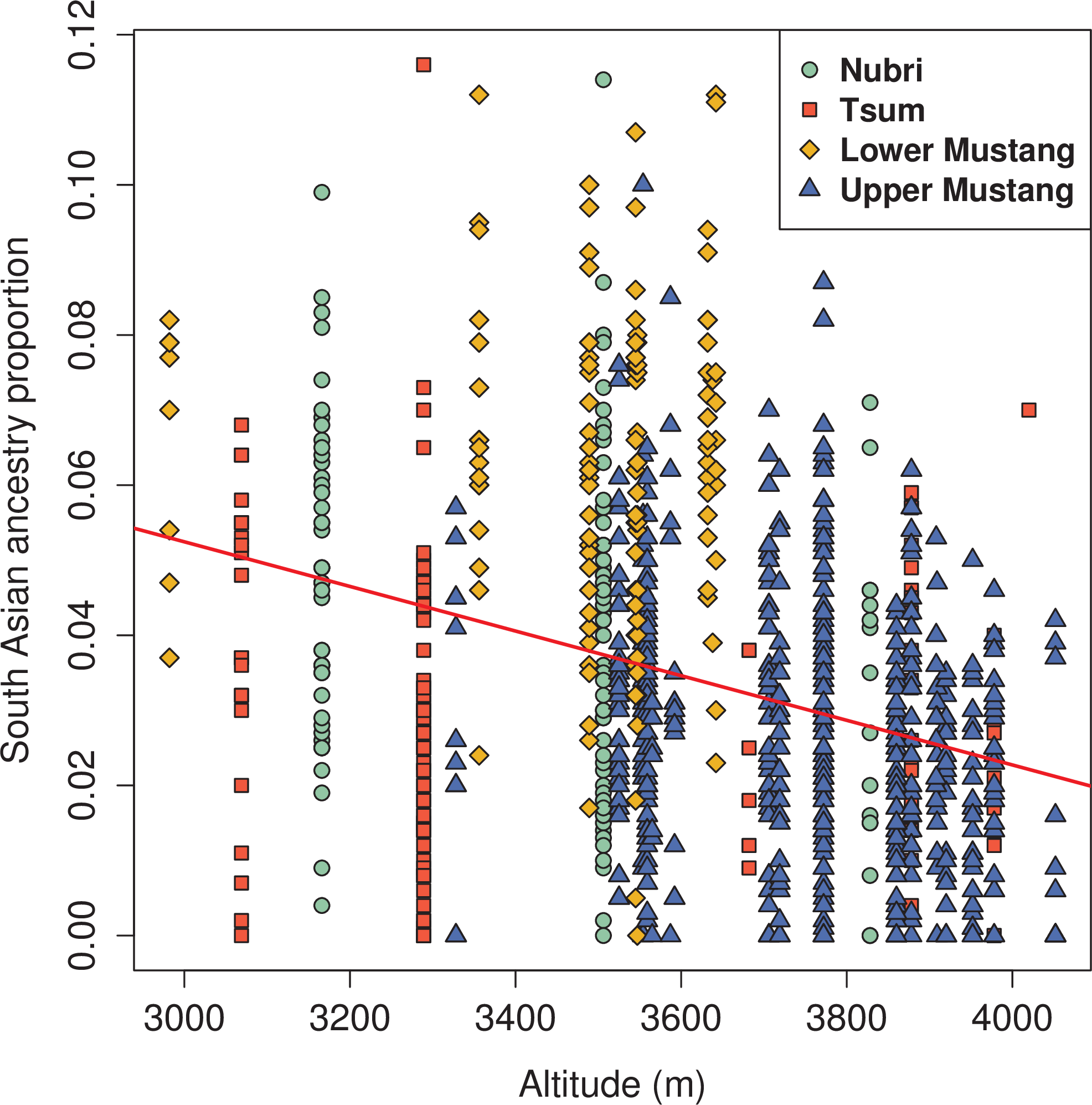

Supplement: S11 Fig — Each color-filled symbols represents a single individual. The red solid line shows a simple linear regression line. The observed negative correlation is highly significant even after controlling for the sub-district label (effect size β = -1.835×10^−5^, p = 2.89×10^−9^). (TIF) [file pgen.1007650.s011.tif]

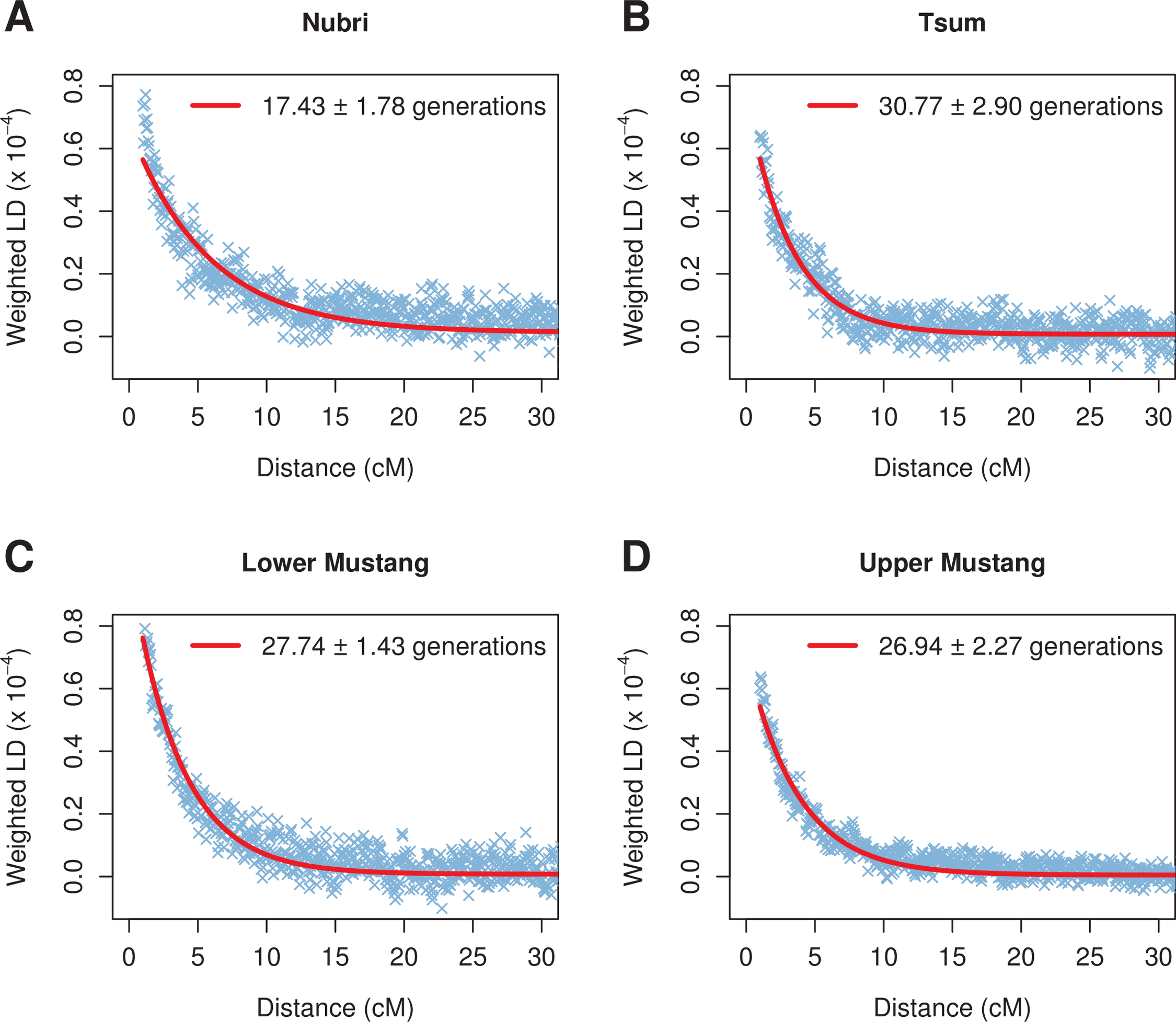

Supplement: S12 Fig — The solid line curves show an exponential fit to the observed weighted admixture LD decay using ALDER. Point estimates ± one standard error estimates are shown in each panel. Standard errors are estimated by leave-one-chromosome-out approach, as implemented in ALDER. (TIF) [file pgen.1007650.s012.tif]
