# Supplementary material for: Detecting past and ongoing natural selection among ethnically Tibetan women at high altitude in Nepal
Source: PLoS Genet. 2018 Sep 6;14(9):e1007650. doi: 10.1371/journal.pgen.1007650 (PMC6143271; doi:10.1371/journal.pgen.1007650)
Supplement: S1 Table — (PDF) [file pgen.1007650.s013.pdf]

S1 Table. Indigenous high-altitude individuals for whole genome sequencing and Illumina array genotyping

A. Whole genome sequencing

Population	Coverage group	Sampling scheme	N	Platform	Coverage	Source
Tibetan	High	Unrelated	2	HiSeq 2000	~30x	[1]
	High	Duo	8	HiSeq 2500	~20x	This study
	Low	Unrelated	27	HiSeq 2500	~5x	This study
Sherpa	High	Unrelated	4	HiSeq 2500	~30x	[1, 2]
	High	Trio	9	HiSeq 2500	~20x	This study
	Low	Unrelated	9	HiSeq 2500	~5x	This study

B. Illumina array genotyping

Population	N	Genotyping Platform	Source
Tibetan	657	HumanCore-12 v1.0A	This study
	344	HumanCore-12 v1.0A, OmniExpress-24 v1.0	This study
Sherpa	103	OmniExpress-24 v1.0	This study
	69	Omni1-Quad	[1]

References

1. Mallick S, Li H, Lipson M, Mathieson I, Gymrek M, Racimo F et al. (2016) The Simons Genome Diversity Project: 300 genomes from 142 diverse populations. *Nature* 538: 201-206.
2. Jeong C, Alkorta-Aranburu G, Basnyat B, Neupane M, Witonsky DB, Pritchard JK et al. (2014) Admixture facilitates genetic adaptations to high altitude in Tibet. *Nat Commun* 5: 3281.
