# Supplementary material for: Detecting past and ongoing natural selection among ethnically Tibetan women at high altitude in Nepal
Source: PLoS Genet. 2018 Sep 6;14(9):e1007650. doi: 10.1371/journal.pgen.1007650 (PMC6143271; doi:10.1371/journal.pgen.1007650)
Supplement: S2 Table — (PDF) [file pgen.1007650.s014.pdf]

S2 Table. A summary of multi-sample variant calling of 59 high-altitude genomes.

A. Types of variants

Type	Subtype	# of SNPs
All		9,742,498
Not present in 1KGP phase 3	All	1,364,150
	Intergenic	746,786
	Intronic	475,302
	UTR	14,719
	Splicing	126
	ncRNA	93,631
	Exonic	13,974
	Exonic - silent	4,784
	Exonic - missense	8,679
	Exonic - nonsense	235

B. Site Frequency Spectrum

# ALT	Proportion (%)
1	29.46
2	24.14
3	11.51
4	7.05
5	4.40
> 5	23.43
