# Supplementary material for: Detecting past and ongoing natural selection among ethnically Tibetan women at high altitude in Nepal
Source: PLoS Genet. 2018 Sep 6;14(9):e1007650. doi: 10.1371/journal.pgen.1007650 (PMC6143271; doi:10.1371/journal.pgen.1007650)
Supplement: S3 Table — (PDF) [file pgen.1007650.s015.pdf]

S3 Table. Phenotypes for genome-wide association analysis

Phenotype		N	nSNPs
**1. Physiological phenotypes**			
Hb	Blood hemoglobin concentration (g/dL)	921	3,507,568
SaO_2	Arterial blood oxygen saturation (%)	921	3,507,568
Pulse	The number of pulses per minute	920	3,508,046
oxyHb	Oxygenated Hb (= Hb × SaO_2 / 100)	921	3,507,568
deoxyHb	Deoxygenated Hb (= Hb - oxyHb)	921	3,507,568
**2. Fertility count phenotypes**			
# of pregnancies		981	3,507,697
# of live births		981	3,507,697
# of children born alive but died < 1 yr		923	3,507,108
# of children surviving at 1 yr but died < 5 yr		849	3,507,585
# of children surviving at 5 yr but died < 15 yr		531	3,506,695
# of children born alive but died < 5 yr		849	3,507,585
# of children born alive but died < 15 yr		532	3,506,123
# of children surviving at 1 yr		923	3,507,108
# of children surviving at 5 yr		849	3,507,585
# of children surviving at 15 yr		531	3,506,695
# of stillbirths		981	3,507,697
# of miscarriages		981	3,507,697
# of twin births		981	3,507,697
A woman's age at her first childbirth		972	3,507,330
A woman's age at her last pregnancy		958	3,507,463
**3. Fertility proportion phenotypes**			
Proportion of live births among pregnancies		981	3,507,697
Proportion of stillbirths among pregnancies		981	3,507,697
Proportion of miscarriages among pregnancies		981	3,507,697
Proportion of children born alive but died < 1 yr		923	3,507,108
Proportion of children born alive but died < 5 yr		849	3,507,585
Proportion of children born alive but died < 15 yr		532	3,506,123
Proportion of children surviving at 1 yr but died < 5 yr		849	3,507,585
Proportion of children surviving at 5 yr but died < 15 yr		531	3,507,249

4. Covariates

Hb ~ sub + age + SaO_2 + ft SaO_2 ~ sub + age + alt + Hb + Hb^2 + ft

Pulse ~ sub + Hb + SaO_2 + ft   oxyHb ~ sub + age + age^2 + ft   deoxyHb ~ sub + age + alt + ft

Fertility counts and proportions ~ sub + age + ct + CM

Sub = subdistrict label (4 groups); alt = altitude of residence
ft = fingertip temperature; ct = use of contraception; CM = continuously married
