# Supplementary material for: Detecting past and ongoing natural selection among ethnically Tibetan women at high altitude in Nepal
Source: PLoS Genet. 2018 Sep 6;14(9):e1007650. doi: 10.1371/journal.pgen.1007650 (PMC6143271; doi:10.1371/journal.pgen.1007650)
Supplement: S4 Table — POS column is for the genomic position in hg19. REF, ALT and DEN columns show reference, alternative and Denisovan alleles, respectively. TBN and CHB present alternative allele frequencies in our Tibetan data and 1KGP CHB, respectively. Effect size estimates β’s are calculated per alternative allele. SNPs with a derived allele shared between Tibetans and Denisovan are marked in bold face. (PDF) [file pgen.1007650.s016.pdf]

S4 Table. The eight *EPAS1* SNPs on chromosome 2 with genome-wide significant association with oxyHb. POS column is for the genomic position in hg19. REF, ALT and DEN columns show reference, alternative and Denisovan alleles, respectively. TBN and CHB present alternative allele frequencies in our Tibetan data and 1KGP CHB, respectively. Effect size estimates β's are calculated per alternative allele. SNPs with a derived allele shared between Tibetans and Denisovan are marked in bold face.

SNP	POS	REF	ALT	DEN	TBN	CHB	β_{oxyHb}	P_{oxyHb}	β_{Hb}	P_{Hb}
**rs374487821**	46571435	G	C	C	0.753	0.005	-0.369	4.00×10^{-8}	-0.378	4.10×10^{-7}
**rs76242811**	46576918	T	C	C	0.749	0.010	-0.374	2.59×10^{-8}	-0.383	1.76×10^{-7}
**rs188801636**	46577251	T	C	C	0.749	0.010	-0.374	1.34×10^{-8}	-0.383	1.76×10^{-7}
**rs375554942**	46579689	A	G	G	0.748	0.010	-0.369	1.95×10^{-8}	-0.380	1.91×10^{-7}
rs189807021	46583581	G	A	G	0.748	0.010	-0.370	1.77×10^{-8}	-0.383	1.65×10^{-7}
**rs372272284**	46584859	A	G	G	0.752	0.010	-0.386	5.71×10^{-9}	-0.396	7.70×10^{-8}
rs150877473	46588019	C	G	C	0.748	0.010	-0.369	2.06×10^{-8}	-0.380	2.18×10^{-7}
rs142826801	46588331	G	C	G	0.745	0.010	-0.371	2.14×10^{-8}	-0.384	1.84×10^{-7}
