# Supplementary material for: Detecting past and ongoing natural selection among ethnically Tibetan women at high altitude in Nepal
Source: PLoS Genet. 2018 Sep 6;14(9):e1007650. doi: 10.1371/journal.pgen.1007650 (PMC6143271; doi:10.1371/journal.pgen.1007650)
Supplement: S6 Table — Tests with p ≤ 0.01 were marked with grey shades. Positive regression coefficients are marked in red color. (PDF) [file pgen.1007650.s018.pdf]

S6 Table. *P*-values for the correlation between fertility and physiological phenotypes. Tests with $p \leq 0.01$ were marked with grey shades. Positive regression coefficients are marked in red color.

Phenotype	Hb	Sat	Pulse	oxyHb	deoxyHb
# of pregnancies	0.398	0.949	2.02×10^{-5}	0.395	0.832
# of live births	0.135	0.996	2.76×10^{-5}	0.142	0.652
# of children born alive but died < 1 yr	0.052	**0.639**	4.84×10^{-3}	0.114	0.219
# of children surviving at 1 yr but died < 5 yr	0.429	**0.598**	0.393	0.561	0.538
# of children surviving at 5 yr but died < 15 yr	0.768	0.891	1.38×10^{-3}	0.707	**0.947**
# of children born alive but died < 5 yr	0.040	**0.454**	0.012	0.109	0.156
# of children born alive but died < 15 yr	0.015	0.663	0.029	0.012	0.597
# of children surviving at 1 yr	0.246	0.524	6.89×10^{-3}	0.138	**0.751**
# of children surviving at 5 yr	0.484	0.384	0.030	0.264	**0.541**
# of children surviving at 15 yr	0.685	**0.564**	0.421	0.836	0.635
# of stillbirths	**0.040**	**0.795**	0.043	**0.034**	**0.686**
# of miscarriages	**0.057**	0.784	**0.769**	**0.074**	**0.458**
# of twin births	**0.747**	**0.587**	0.289	**0.596**	0.744
A woman's age at her first childbirth	**0.255**	0.885	**0.707**	**0.295**	**0.639**
A woman's age at her last pregnancy	0.404	0.737	0.035	0.297	**0.846**
Proportion of live births among pregnancies	2.20×10^{-3}	**0.915**	**0.994**	3.08×10^{-3}	0.335
Proportion of stillbirths among pregnancies	**0.023**	**0.856**	0.203	**0.022**	**0.603**
Proportion of miscarriages among pregnancies	**0.033**	0.774	**0.273**	**0.048**	**0.419**
Proportion of children born alive but died < 1 yr	0.194	**0.446**	0.101	0.430	0.194
Proportion of children born alive but died < 5 yr	0.159	**0.250**	0.217	0.452	0.115
Proportion of children born alive but died < 15 yr	0.075	0.555	0.150	0.056	0.825
Proportion of children surviving at 1 yr but died < 5 yr	0.557	**0.458**	0.832	0.793	0.442
Proportion of children surviving at 5 yr but died < 15 yr	0.761	0.943	2.21×10^{-3}	0.740	0.971
