# Supplementary material for: Detecting past and ongoing natural selection among ethnically Tibetan women at high altitude in Nepal
Source: PLoS Genet. 2018 Sep 6;14(9):e1007650. doi: 10.1371/journal.pgen.1007650 (PMC6143271; doi:10.1371/journal.pgen.1007650)
Supplement: S7 Table — Per-allele selection coefficient for 80% power to detect association in a single SNP test (α = 0.05) was estimated for fertility count phenotypes. No test showed p ≤ 0.01. (PDF) [file pgen.1007650.s019.pdf]

S7 Table. *P* values for the correlation between fertility or fertility proportion phenotypes controlling for covariates and the *EGLN1* and *EPAS1* SNP genotypes. Per-allele selection coefficient for 80% power to detect association in a single SNP test (α = 0.05) was estimated for fertility count phenotypes. No test showed $p \leq 0.01$.

	EGLN1[a]		*EPAS1*[b]	
Phenotype	*P*-value	$s_{80\%\ power}$[c]	*P*-value	$s_{80\%\ power}$
# of pregnancies	0.564	0.066	0.621	0.074
# of live births	0.925	0.068	0.951	0.076
# of children born alive and died < 1 yr	0.119	0.146	0.046	0.152
# of children died ≥ 1 yr and < 5 yrs	0.888	0.197	0.813	0.203
# of children died ≥ 5 yr and < 15 yrs	0.095	0.256	0.083	0.259
# of children died < 5 yrs	0.505	0.126	0.064	0.132
# of children died < 15 yrs	0.045	0.140	0.194	0.147
# of children surviving at 1 yr	0.970	0.068	0.395	0.076
# of children surviving at 5 yr	0.965	0.073	0.307	0.082
# of children surviving at 15 yr	0.599	0.103	0.670	0.117
# of stillbirths	0.053	0.273	0.400	0.274
# of miscarriages	0.111	0.251	0.117	0.254
# of twin births	0.338		0.683	
A woman's age at her first childbirth	0.090		0.346	
A woman's age at her last pregnancy	0.187		0.151	
Proportion of live births among pregnancies	0.020		0.126	
Proportion of stillbirths among pregnancies	0.074		0.451	
Proportion of miscarriages among pregnancies	0.139		0.142	
Proportion of children born alive but died < 1 yr	0.159		0.025	
Proportion of children born alive but died < 5 yr	0.605		0.028	
Proportion of children born alive but died < 15 yr	0.147		0.177	
Proportion of children surviving at 1 yr but died < 5 yr	0.893		0.556	
Proportion of children surviving at 5 yr but died < 15 yr	0.167		0.086	

[a] The number of derived (Tibetan) allele in the *EGLN1* SNP rs186996510
[b] The number of derived (Tibetan) allele in the *EPAS1* SNP rs372272284
[c] Estimated per allele selection coefficient for 80% power with a single test (α = 0.05)
