# Supplementary material for: Detecting past and ongoing natural selection among ethnically Tibetan women at high altitude in Nepal
Source: PLoS Genet. 2018 Sep 6;14(9):e1007650. doi: 10.1371/journal.pgen.1007650 (PMC6143271; doi:10.1371/journal.pgen.1007650)
Supplement: S8 Table — (PDF) [file pgen.1007650.s020.pdf]

S8 Table. Results of polygenic adaptation tests for the GWAS phenotypes

Phenotype	# SNPs	Direction[a]	$P_{frq.diff}$[b]	$P_{overdispersion}$[c]	$P_{outlier}$[d]
**1. Physiological phenotypes**					
Hb	36	-	0.0470	0.0654	0.0000
Hb (no *EPAS1*)	35	-	0.4666	0.2006	0.8460
Hb (European)	91	-	0.2488	0.4334	0.1100
SaO_2	48	+	0.8517	0.5804	0.6464
oxyHb	43	-	0.1016	0.6828	0.0008
oxyHb (no *EPAS1*)	42	-	0.5544	0.6952	0.0656
deoxyHb	45	-	0.0232	0.2478	0.0024
Pulse	52	-	0.0620	0.1570	0.4048
Pulse (European)	123	-	0.0001	0.1020	0.4080
**2. Fertility Counts (all samples)**					
# of pregnancies	37	+	0.4914	0.1598	0.7732
# of live births	40	+	0.5298	0.0284	0.8652
# of children born alive but died < 1 yr	44	-	0.2638	0.4788	0.0940
# of children surviving at 1 yr but died < 5 yr	39	-	0.1344	0.6272	0.0000
# of children surviving at 5 yr but died < 15 yr	45	-	0.0702	0.6494	0.1020
# of children born alive but died < 5 yr	34	-	0.3784	0.4580	0.2060
# of children born alive but died < 15 yr	46	-	0.1159	0.2968	0.0936
# of children surviving at 1 yr	46	+	0.0166	0.0402	0.0100
# of children surviving at 5 yr	45	+	0.4383	0.8132	0.8672
# of children surviving at 15 yr	45	+	0.3128	0.2444	0.4780
# of stillbirths	60	-	0.5398	0.3850	0.3264
# of miscarriages	53	-	0.8412	0.5244	0.0840
# of twin births	47	-	0.1389	0.7576	0.0024
A woman’s age at her first childbirth	35	-	0.4003	0.8180	0.3180
A woman’s age at her last pregnancy	35	+	0.3673	0.4608	0.9920
**3. Fertility proportions (all samples)**					
Proportion of live births among pregnancies	31	+	0.6096	0.6666	0.7820
Proportion of stillbirths among pregnancies	39	-	0.2852	0.6026	0.0004
Proportion of miscarriages among pregnancies	35	-	0.7469	0.0534	0.3604
Proportion of children born alive but died < 1 yr	41	-	0.7819	0.1354	0.2956
Proportion of children born alive but died < 5 yr	32	-	0.9329	0.0848	0.5504
Proportion of children born alive but died < 15 yr	42	-	0.5430	0.1498	0.4452
Proportion of children surviving at 1 yr but died < 5 yr	35	-	0.4650	0.5392	0.8728
Proportion of children surviving at 5 yr but died < 15 yr	40	-	0.2881	0.9640	0.5504

(*Continued in the next page*)

S8 Table. (*Continued from the previous page*)

Phenotype	# SNPs	Direction[a]	$P_{frq.diff}$[b]	$P_{overdispersion}$[c]	$P_{outlier}$[d]
**4. Fertility Counts (CM subset)**					
# of pregnancies	49	+	0.0132	0.6232	0.2892
# of live births	43	+	0.0016	0.0282	0.0020
# of children born alive but died < 1 yr	40	-	0.0121	0.0746	0.0000
# of children surviving at 1 yr but died < 5 yr	42	-	0.7543	0.7786	0.7896
# of children surviving at 5 yr but died < 15 yr	68	-	0.3043	0.5702	0.0256
# of children born alive but died < 5 yr	32	-	0.4068	0.6558	0.1668
# of children born alive but died < 15 yr	50	-	0.0236	0.2432	0.0000
# of children surviving at 1 yr	44	+	0.6344	0.2674	0.1028
# of children surviving at 5 yr	50	+	0.4294	0.3374	0.4912
# of children surviving at 15 yr	72	+	0.2506	0.5352	0.6644
# of stillbirths	85	-	0.8547	0.3146	0.6988
# of miscarriages	91	-	0.0243	0.3632	0.0000
# of twin births	59	-	0.0210	0.6340	0.0000
A woman's age at her first childbirth	60	-	0.5764	0.0714	0.0000
A woman's age at her last pregnancy	48	+	0.7874	0.2622	0.6476
**5. Fertility proportions (CM subset)**					
Proportion of live births among pregnancies	38	+	0.4013	0.1380	0.1624
Proportion of stillbirths among pregnancies	44	-	0.2299	0.3822	0.0008
Proportion of miscarriages among pregnancies	35	-	0.3929	0.1486	0.3480
Proportion of children born alive but died < 1 yr	44	-	0.2765	0.6604	0.2812
Proportion of children born alive but died < 5 yr	38	-	0.5171	0.9830	0.0740
Proportion of children born alive but died < 15 yr	39	-	0.1015	0.3980	0.0224
Proportion of children surviving at 1 yr but died < 5 yr	52	-	0.8736	0.4502	0.2332
Proportion of children surviving at 5 yr but died < 15 yr	44	-	0.6447	0.5886	0.0548

[a] Assumed direction of positive selection for each phenotype
[b] One-sided empirical *p*-value for the mean frequency difference test
[c] *P*-value for the overdispersion test
[d] *P*-value for the outlier test of Tibetans and the Sherpa
[e] Results with the continuously married subset of individuals
