# Supplementary material for: Detecting past and ongoing natural selection among ethnically Tibetan women at high altitude in Nepal
Source: PLoS Genet. 2018 Sep 6;14(9):e1007650. doi: 10.1371/journal.pgen.1007650 (PMC6143271; doi:10.1371/journal.pgen.1007650)
Supplement: S10 Table — Sherpas from the Khumbu region and a South Asian population are used as sources. All four sub-district groups are adequately approximated by the two-way model (P2way ≥ 0.05). Coef1, Coef2 and SE columns represent estimated ancestry proportions from Ref1 (Sherpa) and Ref2 (South Asian), as well as their associated standard errors. Although the South Asian ancestry proportion is small (2.5–6.2%), it is necessary to explain the Himalayan Tibetans, as shown by the insufficiency of the Sherpa-only model (P1way << 0.05). (PDF) [file pgen.1007650.s022.pdf]

S10 Table. Two-way admixture modeling of the Himalayan Tibetans using the qpAdm program. Sherpas from the Khumbu region and a South Asian population are used as sources. All four sub-district groups are adequately approximated by the two-way model ($P_{2way} \geq 0.05$). $Coef_1$, $Coef_2$ and SE columns represent estimated ancestry proportions from Ref_1 (Sherpa) and Ref_2 (South Asian), as well as their associated standard errors. Although the South Asian ancestry proportion is small (2.5-6.2%), it is necessary to explain the Himalayan Tibetans, as shown by the insufficiency of the Sherpa-only model ($P_{1way} << 0.05$).

Target	Ref_1	Ref_2	P_{2way}	P_{1way}	$Coef_1$	$Coef_2$	SE
Nubri	Sherpa	Brahui	0.853	4.92×10^{-27}	0.961	0.039	0.003
		Kalash	0.826	5.94×10^{-27}	0.959	0.041	0.004
		Pathan	0.888	3.57×10^{-27}	0.958	0.042	0.004
Tsum	Sherpa	Brahui	0.385	3.02×10^{-12}	0.974	0.026	0.004
		Kalash	0.263	6.14×10^{-12}	0.973	0.027	0.004
		Pathan	0.310	4.22×10^{-12}	0.972	0.028	0.004
Lower Mustang	Sherpa	Brahui	0.618	2.82×10^{-59}	0.943	0.057	0.003
		Kalash	0.280	1.54×10^{-58}	0.940	0.060	0.004
		Pathan	0.505	2.87×10^{-59}	0.938	0.062	0.004
Upper Mustang	Sherpa	Brahui	0.487	3.31×10^{-16}	0.975	0.025	0.003
		Kalash	0.380	5.81×10^{-16}	0.974	0.026	0.003
		Pathan	0.410	3.93×10^{-16}	0.973	0.027	0.003
