# Supplementary material for: Detecting past and ongoing natural selection among ethnically Tibetan women at high altitude in Nepal
Source: PLoS Genet. 2018 Sep 6;14(9):e1007650. doi: 10.1371/journal.pgen.1007650 (PMC6143271; doi:10.1371/journal.pgen.1007650)
Supplement: S11 Table — We used effect size estimates from multiple studies, ranging from 0.386 to 1.676 g/dL per allele. Power was calculated using the observed allele frequency (maf = 0.336), sample size (n = 649) and standard deviation of Hb residuals after regressing out covariates (1.330 g/dL), assuming a single test (α = 0.05). (PDF) [file pgen.1007650.s023.pdf]

S11 Table. Estimates of power to detect an association between the *EGLN1* SNP rs186996510 and Hb in our data set. We used effect size estimates from multiple studies, ranging from 0.386 to 1.676 g/dL per allele. Power was calculated using the observed allele frequency (maf = 0.336), sample size (n=649) and standard deviation of Hb residuals after regressing out covariates (1.330 g/dL), assuming a single test ($\alpha = 0.05$).

Source	Effect size (g/dL/allele)	Power
Simonson et al. (mixed gender) [1]	1.676	1.000
Xiang et al. (male only) [2]	0.620	1.000
This study (top *EPAS1* SNP rs372272284)	0.386	0.999
An estimate for 99% power	0.332	0.990
An estimate for 95% power	0.280	0.950
An estimate for 90% power	0.252	0.900
An estimate for 80% power	0.218	0.800

References

1. Simonson TS, Yang Y, Huff CD, Yun H, Qin G, Witherspoon DJ et al. (2010) Genetic evidence for high-altitude adaptation in Tibet. *Science* 329: 72-75.
2. Xiang K, Peng Y, Yang Z, Zhang X, Cui C, Zhang H et al. (2013) Identification of a Tibetan-specific mutation in the hypoxic gene *EGLN1* and its contribution to high-altitude adaptation. *Mol Biol Evol* 30: 1889-1898.
