# Supplementary material for: Detecting past and ongoing natural selection among ethnically Tibetan women at high altitude in Nepal
Source: PLoS Genet. 2018 Sep 6;14(9):e1007650. doi: 10.1371/journal.pgen.1007650 (PMC6143271; doi:10.1371/journal.pgen.1007650)
Supplement: S12 Table — For each phenotype, we present the number of individuals included in the main GWAS (nind), the number of unrelated individuals (nunrelated), and Pearson’s correlation coefficient across overlapping SNPs (r). For fertility phenotypes, we present results both for all-sample set and for the continuously married subset. (PDF) [file pgen.1007650.s024.pdf]

S12 Table. Correlation of GWAS $-\log_{10}(p)$ between the main GWAS run including relatives and the one including unrelated individuals only. For each phenotype, we present the number of individuals included in the main GWAS (n_{ind}), the number of unrelated individuals ($n_{unrelated}$), and Pearson's correlation coefficient across overlapping SNPs (*r*). For fertility phenotypes, we present results both for all-sample set and for the continuously married subset.

Phenotype	All-sample set			Continuously married		
**1. Physiological phenotypes**	n_{ind}	$n_{unrelated}$	*r*	n_{ind}	$n_{unrelated}$	*r*
Hb	921	339	0.210			
SaO_2	921	339	0.343			
Pulse	920	339	0.328			
oxyHb	921	339	0.213			
deoxyHb	921	339	0.367			
**2. Fertility count phenotypes**						
# of pregnancies	981	344	0.345	585	272	0.425
# of live births	981	344	0.345	585	272	0.429
# of children born alive but died < 1 yr	923	331	0.363	555	262	0.409
# of children surviving at 1 yr but died < 5 yr	849	306	0.293	513	241	0.342
# of children surviving at 5 yr but died < 15 yr	531	190	0.349	319	150	0.434
# of children born alive but died < 5 yr	849	306	0.358	513	241	0.407
# of children born alive but died < 15 yr	532	190	0.312	320	150	0.378
# of children surviving at 1 yr	923	331	0.368	555	262	0.472
# of children surviving at 5 yr	849	306	0.363	513	241	0.459
# of children surviving at 15 yr	531	190	0.377	319	150	0.459
# of stillbirths	981	344	0.336	585	272	0.439
# of miscarriages	981	344	0.355	585	272	0.441
# of twin births	981	344	0.301	585	272	0.431
A woman's age at her first childbirth	972	344	0.260	581	272	0.445
A woman's age at her last pregnancy	958	344	0.330	575	272	0.445
**3. Fertility proportion phenotypes**						
Proportion of live births among pregnancies	981	344	0.348	585	272	0.440
Proportion of stillbirths among pregnancies	981	344	0.318	585	272	0.415
Proportion of miscarriages among pregnancies	981	344	0.300	585	272	0.409
Proportion of children born alive but died < 1 yr	923	331	0.352	555	262	0.454
Proportion of children born alive but died < 5 yr	849	306	0.346	513	241	0.451
Proportion of children born alive but died < 15 yr	532	190	0.387	320	150	0.464
Proportion of children surviving at 1 yr but died < 5 yr	849	306	0.329	513	241	0.449
Proportion of children surviving at 5 yr but died < 15 yr	531	190	0.384	319	150	0.440
