# Supplementary material for: Detecting past and ongoing natural selection among ethnically Tibetan women at high altitude in Nepal
Source: PLoS Genet. 2018 Sep 6;14(9):e1007650. doi: 10.1371/journal.pgen.1007650 (PMC6143271; doi:10.1371/journal.pgen.1007650)
Supplement: S1 Text — (PDF) [file pgen.1007650.s025.pdf]

S1 Text. Limited South Asian admixture in ethnic Tibetans from the Himalayan valleys in Nepal

The southern slope of the Himalayas is a zone of contact and admixture between various populations of South and East Asian origins [1, 2]. Although previous studies found that recent genetic contribution from South Asian gene pools rapidly decreases when moving from low to high altitudes [1, 2], presence and magnitude of South Asian ancestry in the ethnic Tibetan groups we studied here have not been studied so far. To characterize South Asian admixture in the Himalayan Tibetans in our study, we first compiled a genome-wide genotype data of world-wide populations from published studies, either array-genotyped on Illumina platforms [3-11] or whole-genome sequenced [5, 7, 8, 12-28]. We intersected SNPs between different platforms and filtered low-quality SNPs and individuals (SNPs and individuals with > 5% missing rate per data set) using PLINK v1.9 [29], resulting in 3,256 individuals and 224,268 autosomal SNPs, including our new data of 991 individuals (S9 Table).

We first performed a principal component analysis (PCA) of Eurasian populations using the smartpca v16000 program in the EIGENSOFT v7.2 package [30]. Because PCA is sensitive to sample size and our Himalayan Tibetans have much bigger sample sizes than the rest of groups in the panel, we calculated PCs without Himalayan Tibetan individuals; instead, we projected them onto PCs calculated from a published panel of 1,295 Eurasian individuals using the "*lsqproject: YES*" option (S9 Table). This approach also provides an unbiased way of comparing related individuals in PCA. We repeated the same analysis using 357 East Asian and Siberian individuals with no detectable Western Eurasian ancestry (S9 Table).

Eurasian PCA results show that all of the Himalayan Tibetans form a cluster close to other East Asians but far from South Asians (S7 Fig). In the East Asian PCA, they fall on next to present-day Tibetans from near Lhasa (S8 Fig). These results show that there is no large-scale South Asian admixture in the Himalayan Tibetans residing at 3,000 m or higher in altitude.

We calculated outgroup-f_3 statistic of the form f_3(Mbuti; Himalayan Tibetan, world-wide), a measure of shared genetic drift between Himalayan Tibetans and world-wide populations since their split from a common outgroup, Central African rainforest hunter-gatherer Mbuti, for each Himalayan Tibetan group (Nubri, Tsum, Lower Mustang and Upper Mustang). For all four Himalayan groups, Tibetans from Lhasa ("Tibetan") [11] and Sherpas from the Khumbu region ("Sherpa") [10] show the highest outgroup-f_3 values (S9 Fig). We further tested if the Himalayan Tibetans have extra allele sharing with South Asians by calculating f_4(Mbuti, South Asian; Tibetan/Sherpa, Himalayan). Nubri and Lower Mustang show significantly positive f_4 statistics ($f_4 \geq 3$ standard errors, SEs; S9 Fig). The other two, Tsum and Upper Mustang, show positive statistics but within statistical noise (< 3 SEs). Using the qpAdm program, a generalization of f_4 statistic that tests symmetry between the target and the admixture model against

multiple outgroups [20], we find that Sherpa + Pathan provides a good fit to the Himalayan Tibetans with 2.8-6.2% South Asian contribution (χ^2 $p \geq 0.310$; S10 Table). In line with significant positive f_4 statistic with South Asians and other Western Eurasians, the Himalayan Tibetans cannot be modeled as a sister group of Sherpa without the above estimated small amount of South Asian contribution (χ^2 $p \leq 4.22\times10^{-12}$). We used qp3Pop (v400), qpDstat (v711) and qpAdm (v632) programs from the ADMIXTOOLS v3.0 package [31]. For the qpAdm analysis, we used eight outgroups: Mbuti (n=13), Onge (n=2), Dai (n=10), Chukchi (n=12), Nganasan (n=12), Neolithic Anatolian farmers ("Anatolia_N"; n=23) [20], Western European Mesolithic hunter-gatherers ("WHG"; n=3) [15, 21] and Eneolithic Yamnaya individuals from Samara ("Yamnaya_Samara"; n=9) [22].

We applied the same qpAdm model for each individual separately to estimate South Asian admixture proportion without being affected by sample size or population-specific drift. Per-individual South Asian ancestry proportion ranges 0% to 11.6%, with the mean of each group matching with group-based estimates (S10 Fig). South Asian ancestry proportion is negatively correlated with altitude even after taking into account sub-region labels ($p = 2.89\times10^{-9}$; S11 Fig).

Admixture dates are estimated to be between 17 and 31 generations ago (505-892 years before present, using 29 years per generation; S12 Fig) using weighted admixture LD-based method implemented in ALDER v1.3 [32]. We used Sherpas and Pathans as two references and minimum distance of 1.0 cM for fitting the exponential curve.

References

1. Gnecchi-Ruscone GA, Jeong C, De Fanti S, Sarno S, Trancucci M, Gentilini D, et al. The genomic landscape of Nepalese Tibeto-Burmans reveals new insights into the recent peopling of Southern Himalayas. *Sci Rep* 2017; 7: 15512.
2. Cole AM, Cox S, Jeong C, Petousi N, Aryal DR, Droma Y, et al. Genetic structure in the Sherpa and neighboring Nepalese populations. *BMC Genomics* 2017; 18: 102.
3. Li JZ, Absher DM, Tang H, Southwick AM, Casto AM, Ramachandran S, et al. Worldwide human relationships inferred from genome-wide patterns of variation. *Science* 2008; 319: 1100-1104.
4. Fedorova SA, Reidla M, Metspalu E, Metspalu M, Rootsi S, Tambets K, et al. Autosomal and uniparental portraits of the native populations of Sakha (Yakutia): implications for the peopling of Northeast Eurasia. *BMC Evol Biol* 2013; 13: 127.
5. Rasmussen M, Li Y, Lindgreen S, Pedersen JS, Albrechtsen A, Moltke I, et al. Ancient human genome sequence of an extinct Palaeo-Eskimo. *Nature* 2010; 463: 757-762.
6. Yunusbayev B, Metspalu M, Metspalu E, Valeev A, Litvinov S, Valiev R, et al. The genetic legacy of the expansion of Turkic-speaking nomads across Eurasia. *PLoS Genet* 2015; 11: e1005068.
7. Raghavan M, Skoglund P, Graf KE, Metspalu M, Albrechtsen A, Moltke I, et al. Upper Palaeolithic Siberian genome reveals dual ancestry of Native Americans. *Nature* 2014; 505: 87-91.

8. Raghavan M, DeGiorgio M, Albrechtsen A, Moltke I, Skoglund P, Korneliussen TS, et al. The genetic prehistory of the New World Arctic. *Science* 2014; 345: 1255832.
9. Raghavan M, Steinrücken M, Harris K, Schiffels S, Rasmussen S, DeGiorgio M, et al. Genomic evidence for the Pleistocene and recent population history of Native Americans. *Science* 2015; 349: aab3884.
10. Jeong C, Alkorta-Aranburu G, Basnyat B, Neupane M, Witonsky DB, Pritchard JK, et al. Admixture facilitates genetic adaptations to high altitude in Tibet. *Nat Commun* 2014; 5: 3281.
11. Wang B, Zhang Y-B, Zhang F, Lin H, Wang X, Wan N, et al. On the origin of Tibetans and their genetic basis in adapting high-altitude environments. *PLoS One* 2011; 6: e17002.
12. Mallick S, Li H, Lipson M, Mathieson I, Gymrek M, Racimo F, et al. The Simons Genome Diversity Project: 300 genomes from 142 diverse populations. *Nature* 2016; 538: 201-206.
13. Allentoft ME, Sikora M, Sjogren K-G, Rasmussen S, Rasmussen M, Stenderup J, et al. Population genomics of Bronze Age Eurasia. *Nature* 2015; 522: 167-172.
14. Fu Q, Li H, Moorjani P, Jay F, Slepchenko SM, Bondarev AA, et al. Genome sequence of a 45,000-year-old modern human from western Siberia. *Nature* 2014; 514: 445-449.
15. Fu Q, Posth C, Hajdinjak M, Petr M, Mallick S, Fernandes D, et al. The genetic history of Ice Age Europe. *Nature* 2016; 534: 200-205.
16. Haber M, Doumet-Serhal C, Scheib C, Xue Y, Danecek P, Mezzavilla M, et al. Continuity and admixture in the last five millennia of Levantine history from ancient Canaanite and present-day Lebanese genome sequences. *Am J Hum Genet* 2017; 101: 274-282.
17. Jones ER, Gonzalez-Fortes G, Connell S, Siska V, Eriksson A, Martiniano R, et al. Upper Palaeolithic genomes reveal deep roots of modern Eurasians. *Nat Commun* 2015; 6: 8912.
18. Kılınç Gülşah M, Omrak A, Özer F, Günther T, Büyükkarakaya Ali M, Bıçakçı E, et al. The demographic development of the first farmers in Anatolia. *Curr Biol* 2016; 26: 2659-2666.
19. Lazaridis I, Mittnik A, Patterson N, Mallick S, Rohland N, Pfrengle S, et al. Genetic origins of the Minoans and Mycenaeans. *Nature* 2017; 548: 214-218.
20. Lazaridis I, Nadel D, Rollefson G, Merrett DC, Rohland N, Mallick S, et al. Genomic insights into the origin of farming in the ancient Near East. *Nature* 2016; 536: 419-424.
21. Lazaridis I, Patterson N, Mittnik A, Renaud G, Mallick S, Kirsanow K, et al. Ancient human genomes suggest three ancestral populations for present-day Europeans. *Nature* 2014; 513: 409-413.
22. Mathieson I, Lazaridis I, Rohland N, Mallick S, Patterson N, Roodenberg SA, et al. Genome-wide patterns of selection in 230 ancient Eurasians. *Nature* 2015; 528: 499-503.
23. Rasmussen M, Anzick SL, Waters MR, Skoglund P, DeGiorgio M, Stafford Jr TW, et al. The genome of a Late Pleistocene human from a Clovis burial site in western Montana. *Nature* 2014; 506: 225-229.
24. Rasmussen M, Sikora M, Albrechtsen A, Korneliussen TS, Moreno-Mayar JV, Poznik GD, et al. The ancestry and affiliations of Kennewick Man. *Nature* 2015; 523: 455-458.
25. Saag L, Varul L, Scheib CL, Stenderup J, Allentoft ME, Saag L, et al. Extensive farming in Estonia started through a sex-biased migration from the Steppe. *Curr Biol* 2017; 27: 2185-2193. e2186.
26. Siska V, Jones ER, Jeon S, Bhak Y, Kim H-M, Cho YS, et al. Genome-wide data from two early Neolithic East Asian individuals dating to 7700 years ago. *Sci Adv* 2017; 3: e1601877.
27. Unterländer M, Palstra F, Lazaridis I, Pilipenko A, Hofmanová Z, Groß M, et al. Ancestry and demography and descendants of Iron Age nomads of the Eurasian Steppe. *Nat Commun* 2017; 8: 14615.
28. Yang MA, Gao X, Theunert C, Tong H, Aximu-Petri A, Nickel B, et al. 40,000-Year-Old Individual from Asia Provides Insight into Early Population Structure in Eurasia. *Curr Biol* 2017; 27: 3202-3208.e3209.
29. Chang CC, Chow CC, Tellier L, Vattikuti S, Purcell SM and Lee JJ. Second-generation PLINK: rising to the challenge of larger and richer datasets. *Gigascience* 2015; 4: 7.

30. Patterson N, Price AL and Reich D. Population structure and eigenanalysis. *PLoS Genet* 2006; 2: e190.
31. Patterson N, Moorjani P, Luo Y, Mallick S, Rohland N, Zhan Y, et al. Ancient admixture in human history. *Genetics* 2012; 192: 1065-1093.
32. Loh P-R, Lipson M, Patterson N, Moorjani P, Pickrell JK, Reich D, et al. Inferring admixture histories of human populations using linkage disequilibrium. *Genetics* 2013; 193: 1233-1254.
