# Supplementary material for: Detecting past and ongoing natural selection among ethnically Tibetan women at high altitude in Nepal
Source: PLoS Genet. 2018 Sep 6;14(9):e1007650. doi: 10.1371/journal.pgen.1007650 (PMC6143271; doi:10.1371/journal.pgen.1007650)
Supplement: S2 Text — (PDF) [file pgen.1007650.s026.pdf]

S2 Text. No genetic association between the nonsynonymous *EGLN1* SNPs and Hb phenotypes

Two nonsynonymous variants in the *EGLN1* gene, rs12097901 and rs186996510, were previously shown to harbor strong signatures of selective sweep [1, 2]. An early study reported an association of the *EGLN1* haplotype (defined by a combination of three SNPs; rs2275279, rs2790859 and rs961154) with lower Hb in a sample of mixed gender with large effect size estimate 1.676 g/dL per allele [3]. However, two more recent studies, each with more than 500 Tibetan participants, reported that the derived allele of rs186996510 was associated with lower Hb only in males [1, 4]. Similarly, an analysis of *EGLN1* SNPs in 3,008 Tibetans did not detect a significant association with Hb, and reported a larger effect size for males compared to females [5]. Consistent with the more recent evidence, we did not detect a significant association at either of the two nonsynonymous SNPs with Hb ($p \geq 0.224$) or with oxyHb ($p \geq 0.268$) in our Tibetan sample, which includes only females. No association was detected when we either added menopause status, a female-specific covariate of Hb, as an additional covariate or confined our analysis to post-menopausal women ($p \geq 0.641$), thus excluding it as an explanation for the lack of association in females. We calculated that our power to detect an association with a single test ($\alpha = 0.05$) for the observed allele frequency of rs186996510 (0.336) and our sample size (N = 649) was ≥ 99% for an effect size as low as 0.332 g/dL per allele, which is well below previous estimates (S11 Table). The reasons for the inconsistent findings regarding an association of *EGLN1*, an oxygen sensor in the oxygen homeostasis pathway, and hemoglobin concentration are unknown.

A recent study suggested an interaction between the effects of *EGLN1* and *EPAS1* SNPs on Hb levels [6]. However, our data showed no significant interaction between the *EPAS1* and *EGLN1* SNPs (rs372272284 and rs186996510, respectively) in the association with Hb ($p = 0.613$).

References

1. Xiang K, Peng Y, Yang Z, Zhang X, Cui C, Zhang H et al. (2013) Identification of a Tibetan-specific mutation in the hypoxic gene *EGLN1* and its contribution to high-altitude adaptation. *Mol Biol Evol* 30: 1889-1898.
2. Lorenzo FR, Huff C, Myllymaki M, Olenchock B, Swierczek S, Tashi T et al. (2014) A genetic mechanism for Tibetan high-altitude adaptation. *Nat Genet* 46: 951-956.
3. Simonson TS, Yang Y, Huff CD, Yun H, Qin G, Witherspoon DJ et al. (2010) Genetic evidence for high-altitude adaptation in Tibet. *Science* 329: 72-75.
4. Peng Y, Cui C and He Y (2017) Down-regulation of *EPAS1* transcription and genetic adaptation of Tibetans to high-altitude hypoxia. *Mol Biol Evol* 34: 818-830.
5. Yang J, Jin Z-B, Chen J, Huang X-F, Li X-M, Liang Y-B et al. (2017) Genetic signatures of high-altitude adaptation in Tibetans. *Proc Natl Acad Sci USA* 114: 4189-4194.
6. Tashi T, Reading NS, Wuren T, Zhang X, Moore LG, Hu H et al. (2017) Gain-of-function *EGLN1* prolyl hydroxylase (PHD2 D4E: C127S) in combination with *EPAS1* (HIF-2α) polymorphism lowers hemoglobin concentration in Tibetan highlanders. *J Mol Med* 95: 665-670.
