# Supplementary material for: Detecting past and ongoing natural selection among ethnically Tibetan women at high altitude in Nepal
Source: PLoS Genet. 2018 Sep 6;14(9):e1007650. doi: 10.1371/journal.pgen.1007650 (PMC6143271; doi:10.1371/journal.pgen.1007650)
Supplement: S3 Text — (PDF) [file pgen.1007650.s027.pdf]

S3 Text. Genomic signatures of recent selective sweeps in Tibetans

To summarize genomic signatures of recent selective sweeps in Tibetans, we calculated the population branch statistic (PBS) [1] across the Tibetan genome. This statistic measures the extent of allele frequency divergence on the lineage leading to a test population, in this case Tibetans. We used the 344 unrelated Tibetans genotyped using the Illumina OmniExpress array. For the comparison group and outgroup, we used 1KGP phase 3 CHB and CEU (CEPH Utah residents with Northern and Western European ancestry) respectively. We calculated PBS following Weir and Cockerham's definition of pairwise F_{ST} [2] using a custom python script for markers with maf ≥ 0.05 in either Tibetans or CHB. Only female individuals from the 1KGP data were used to calculate statistics for the X chromosome. After calculating PBS, we summarized the signal for 100 kb windows sliding by 25 kb, by calculating a pseudo-binomial *p*-value *P* defined as:

$$P = \Pr(X \geq n_{top});\ X \sim \text{Binomial}(n_{total}, p = 0.001)$$

where n_{top} and n_{total} represent the number of global top 0.1% SNPs and the number of total SNPs in each window. Cutoffs for the top 0.1% PBS value were calculated separately for the autosomes and the X chromosome. Consistent with previous studies, the *EPAS1* (PBS = 1.073, rs73926264) and *EGLN1* (PBS = 0.797, rs186996510) loci harbored the highest PBS values (S5 Table). We did not find additional genes with high PBS score and strong functional implication.

References

1. Yi X, Liang Y, Huerta-Sanchez E, Jin X, Cuo ZXP, Pool JE et al. (2010) Sequencing of 50 human exomes reveals adaptation to high altitude. *Science* 329: 75-78.
2. Weir BS and Cockerham CC (1984) Estimating F-statistics for the analysis of population structure. *Evolution* 38: 1358-1370.
